# Identification of social relation within pedestrian dyads

**DOI:** 10.1371/journal.pone.0223656

**Published:** 2019-10-17

**Authors:** Zeynep Yucel, Francesco Zanlungo, Claudio Feliciani, Adrien Gregorj, Takayuki Kanda

**Affiliations:** 1 Department of Computer Science, Okayama University, Okayama, Japan; 2 Intelligent Robotics and Communication Laboratory, ATR, Kyoto, Japan; 3 Research Center for Advanced Science and Technology, The University of Tokyo, Tokyo, Japan; 4 Department of Social Informatics, Kyoto University, Kyoto, Japan; Universita degli Studi di Pisa, ITALY

## Abstract

This study focuses on social pedestrian groups in public spaces and makes an effort to identify the type of social relation between the group members. As a first step for this identification problem, we focus on dyads (i.e. 2 people groups). Moreover, as a mutually exclusive categorization of social relations, we consider the domain-based approach of Bugental, which precisely corresponds to social relations of colleagues, couples, friends and families, and identify each dyad with one of those relations. For this purpose, we use anonymized trajectory data and derive a set of *observables* thereof, namely, inter-personal distance, group velocity, velocity difference and height difference. Subsequently, we use the probability density functions (pdf) of these observables as a tool to understand the nature of the relation between pedestrians. To that end, we propose different ways of using the pdfs. Namely, we introduce a probabilistic Bayesian approach and contrast it to a functional metric one and evaluate the performance of both methods with appropriate assessment measures. This study stands out as the first attempt to automatically recognize social relation between pedestrian groups. Additionally, in doing that it uses completely anonymous data and proves that social relation is still possible to recognize with a good accuracy without invading privacy. In particular, our findings indicate that significant recognition rates can be attained for certain categories and with certain methods. Specifically, we show that a very good recognition rate is achieved in distinguishing colleagues from leisure-oriented dyads (families, couples and friends), whereas the distinction between the leisure-oriented dyads results to be inherently harder, but still possible at reasonable rates, in particular if families are restricted to parent-child groups. In general, we establish that the Bayesian method outperforms the functional metric one due, probably, to the difficulty of the latter to learn observable pdfs from individual trajectories.

## Introduction and motivation

In the field of crowd dynamics, recently there is a growing interest in analysis of social group motion. Various empirical studies have demonstrated that group motion is shaped as a result of a complex interplay of social elements such as relation and interaction as well as person-specific (i.e. relating individuals) elements such as age or gender [1–4].

This study addresses particularly one of those elements acting on group motion, namely social relation. Specifically, we aim at automatically recognizing the kind of social relation between members of a pedestrian group. In particular, we consider pairs of two pedestrians (i.e. dyads) as the most basic building block of social pedestrian groups [5].

This paper extends our preliminary work, which discriminated two kinds of social relations [3], by covering potentially the entire range of relation categories that occur between moving pedestrian dyads. In this respect, to the best of our knowledge, this work stands out as the first attempt to apply automatic social relation recognition in mobile settings. By this, we mean that in this work we will limit ourselves to recognizing the social relation of groups *while they are walking*.

Members of social groups, independent of their locomotion properties, prefer keeping a reasonably short distance between themselves, i.e. they are characterized by a specific group “proxemics” (a term that refers to the manner in which individuals behave or interact with each other in terms of their personal space and interpersonal distances [6]). According to [7], the proxemics of moving groups is different from the proxemics of standing ones, since the former ones are constrained by the necessity of keeping their “goal” or walking direction in their field of view, a constrain that, for instance, causes dyads to walk in an abreast formation. Although the detection of standing groups is definitely of great theoretical and practical importance, there is not even a complete consensus on “standing pedestrians” being pedestrians in the strict sense [8], and we restrain in this work from any attempt to detect their social relation.

The proposed method and related findings offer potential improvement in various services and systems. For instance, the use of the proposed probabilistic assessment method can facilitate the process of human labeling and extend the amount of information that we can get out of the (tracking) data. In addition, it may help developing motion models for pedestrian groups with different social relations, which is likely to contribute in building more realistic pedestrian simulators with a diverse profile of agents. Moreover, the proposed method can be deployed on autonomous agents (such as assistive robots) in order to equip them with a better understanding of the crowd and in particular of social groups. This may help such agents in providing to pedestrians automatic services matching to their needs or interests. In addition, by accounting for social relation, further insight into crowd level activities can be achieved and used, for instance, for detecting stability, collectiveness, conflict and abnormal and possibly dangerous or illegal behavior [9, 10].

### Background

Human crowds have a heterogeneous composition, and their two fundamental constituents can be regarded as (i) individuals (i.e. people, who are not acquainted or engaged in social interaction with others, and move independently) and (ii) groups (i.e. people, who are engaged in a social relation to one or more pedestrians and move together toward a common goal [11]). Here, the term “groups” refers specifically to “social pedestrian groups”, implicating a pre-existing acquaintance. In other words, we do not consider as groups those people who coincidentally move together for a short time, due to specific crowd dynamics effects (e.g. self-organizing lanes) or coincident interests (common origin or destination). On the contrary, we regard as groups those people who arrive and move in the observed environment together due to their social relation. Of course, these people could still meet and split at given times. Before they meet and after they split, despite still having a social relation, they are not considered as a social group according to this work’s definition. “Splitting” is empirically defined based on the distance probability distribution of interacting pedestrians, as explained below.

Although social pedestrian groups constitute a significant portion of the crowd [12], in the field of crowd dynamics, a detailed analysis on their motion patterns is still not completely attained. A common approach in studying social group motion has been the adaptation of previous individual pedestrian motion models, such as the social force model, cellular automaton, or agent based models, in order to account for group dynamics [5, 13–16]. In particular, the dynamics of two people and three people groups in sparse environments has been performed in [7], while [17] and [18] analyze the dependence of their behavior on an environmental factor (i.e. density) from an empirical and a theoretical standpoint, respectively. These latter studies are of relevance to this work because they focus on pedestrian group behavior in normal conditions (in contrast to emergency or evacuation behavior). Nevertheless, although each of these studies provided an insightful account on group dynamics, they treated groups based only on their most fundamental properties, i.e. being engaged in social relation and moving toward a common goal.

However, it has been shown that pedestrian groups in themselves present variations in locomotion depending on various traits (e.g. age, gender, relation) and states (e.g. engagement in interaction) [1–3, 19]. Based on the results of [7, 17, 18], our work in [1] performs an analysis of four variables (namely, the dependence of group velocity, distance, abreast distance and distance in the direction of motion) for groups of different “intrinsic properties”. Such “intrinsic properties” are the purpose of visit to the environment (work vs. leisure), gender of the group members, age of the group members, height of the group members, and their (social) relation. It is shown that each intrinsic property affects the probability density functions (pdf) of the four variables. Given this complex interplay of social factors, we believe that an understanding of the effect of these factors on motion bears the potential of enhancing existing group dynamics models.

The proposed study focuses on one of the most interesting and challenging of the intrinsic properties, namely social relation. In what follows, we provide an overview of the recent studies and trends in recognition of social relation in various research fields.

### Related work, recent trends and privacy issues

A recent systematic review by Templeton et al. examines 140 studies on collective behavior in crowd modeling (and simulation) [20]. Templeton et al. identify two main stream approaches as “mass of individuals” and “small groups”, where small groups can further be grouped into non-perceptual, perceptual and cognitive groups. This study uses a definition of “groups” similar to the perceptual sub-type of [20].

Many early works on group behavior were focused on empirical analysis of quantitative variables such as walking speed or interpersonal spacing; and the effect of gender, age, mobility, group size etc. on those variables [21, 22]. The inferences from such studies constituted a basis for the recognition of groups [23] and served useful in simulation of their behavior based on empirical observations [24–26]. Our work distinguishes itself from such studies in the sense that rather than discussing the dependency of motion dynamics on group relation or replicating them in a simulation environment, we target automatically identifying one of the underlying social elements, namely social relation, leading to these observations.

From a practical point of view, an analysis of the intrinsic factors listed in the Background section becomes increasingly affordable with the recent developments in sensing technologies and portable gadgets. Namely, there is a rapid proliferation of sensing systems into daily life involving ubiquitous sensor networks and surveillance systems as well as smart watches, smart phones, activity trackers etc., which provide an abundant amount of data for such an analysis. However, one particular medium, namely social networks, has been a popular application domain for the recognition of such relational features.

One of the early works in this field belongs to Wang et al., who recognize kinship between people appearing in the same image (e.g. siblings, husband-wife, mother-child etc.). The follow-up works in this field keep focusing on photo albums and consider a wider range of relations [27–29]. Additionally, they profit from the abundance of social network data by employing more powerful tools such as Deep Neural Networks (DNN) [30–32].

Conventionally, the main application areas of these recognition systems have been user profiling in social networks or customer behavior in online shops for personal recommender systems etc. Indeed, the experiences collected in such settings indicate that personalization may improve service satisfaction. However, there are also numerous surveys indicating users’ concern about their privacy and the collection and use of their personal information [33]. As a matter of fact, this dichotomy of information privacy attitude and actual behavior brought an interesting twist on the application of the above-mentioned recognition systems. Namely, there is a shift from “product recommender systems” towards “privacy advisors”. In particular, detection of such private features are recently used for preventing posting of private data [34–36].

In this respect, taking into consideration such privacy concerns, this study proposes a recognition method for social relation in public spaces employing anonymous data (although it can also utilize anonymous information derived from potentially non-anonymous data such as closed-circuit television (CCTV) footage). The proposed method differs from the existing studies in several respects:

- The proposed method utilizes completely anonymous data and thus minimizes privacy concerns. Moreover, there is no requirement of access to personal devices or authorized participation of users, neither is there a need for a prolonged observation of activities (e.g. posting of many pictures, video footage etc.).
- The anonymous trajectory data can be derived from a variety of commonly used sensor systems (e.g. laser range finders, RGBD sensors etc.). This not only copes with the privacy concerns, but also strongly reduces the number of sensors needed to collect the information as opposed to camera networks etc.
- The proposed method can potentially provide continuous estimation and run online integrated with real-time autonomous systems. The rate of estimation is limited only by the sampling frequency of the sensors. However, since the frame rate of video systems or the sampling frequency of laser range finders are at the levels of 10s per second, potential estimation rate is quite high.

While the proposed implementation relies entirely on anonymous (trajectory) data, due to its ability to operate on feature spaces of arbitrary dimension, the method can potentially be extended to the use of also non-anonymous features.

### Categorization of social relation

Social relation is any kind of relationship between two or more individuals, entailed with (active) involvement of the parties [37]. That being said, we would like to emphasize that social relation is strictly connected to (social) interaction [38].

Although social relation among individuals has been studied by psychologists for a very long time, due to the diversity of the social situations and primary frame of reference, there is no consensus on a universal, concrete and exhaustive list of social relations. However, there do exist several widely accepted categorizations of fundamental forms of social relation. We examined such prominent categorizations in literature and chose the one, which we regard to best reflect relational properties of a social pedestrian group.

In what follows, we provide a brief overview of some notable categorizations of social relation.

- Fiske claims that social relations are constructed and coordinated based on four basic “elementary forms of social relation”, which are (i) communal sharing that assumes members of a social group to be equivalent and undifferentiated; (ii) authority ranking that is a linear ordering in which everyone’s rank can be compared to anyone else, (iii) equality matching that regulates social relation between any two people based on significant differences or imbalances between them; and (iv) market pricing that orients people in relation to ratio values (for instance, ratios of wages, rents etc.) [39].
- Mills and Clark consider relations from a pragmatic standpoint and identify two general types of relationships, where the key difference is the rules and expectations governing the “giving and receiving of benefits” [40]. According to their theory, relations are either exchange relations or communal relations.
- Foa and Foa ground their theory on exchange relations and offer a categorization based on the type of the resources subject to exchange, which can be either love, status, money, goods, service or information [41].
- Bugental proposes a domain-based approach and divides social life into five non-overlapping domains as attachment, hierarchical power, mating, reciprocity and coalitional [42].

Each of the above listed categorizations claims to be (potentially) mutually exclusive (i.e. not overlapping) and collectively exhaustive (i.e. spanning the set of all social relations), in addition to being universal across cultures. Nevertheless, not all of them apply to any setting, and in particular to pedestrians moving in a public space. Namely, social relations which are consequences of a particular action (e.g. receiving, giving) or depend on the environment (e.g. a classroom with potential authority ranking between teacher and pupils) may not occur in our specific setting.

Thus, we evaluate the feasibility of these categorizations with respect to pedestrian behavior and conclude that the approach of Bugental is the most pertinent one [42]. Of course, the definition of Bugental aims at categorizing the more general cases (i.e. beyond pedestrian settings), and yet it provides a direct association to the commonly occurring social relations in mobile settings.

In particular, it delivers a direct association to the social relations discussed in [1]. Namely, the categories of colleagues, families, couples, and friends treated in [1] correspond to the domains of coalitional, attachment, mating and reciprocal, defined by Bugental, respectively. In this respect, we note that the fifth domain, i.e. hierarchical relation, is eliminated since it does not apply to pedestrians in a public space to the full extent. Moreover, the operability of the approach proposed by [42] is supported by its use in various recent social signal processing studies focusing on social relations [43–45].

## Materials and methods

### Data set

The openly available data set used in this study was introduced by [17]. In what follows, for the integrity of the manuscript, we briefly provide relevant information on the data set but refer the interested reader to [17] and [1] for a thorough discussion.

The data set is recorded in an indoor public space over a one year time window using 3D depth sensors with the consent of local authorities and building managers. Posters explaining that an experiment concerning pedestrian tracking was being hold were present in the environment. Experimentation has been reviewed and approved by ATR ethics board with document number 502-1. Using the algorithm of [46], the pedestrians are automatically tracked and their height and position information (on a 2D floor plane) are extracted, which are all available at [47].

Since the main purpose of this study is the recognition of social relation between members of pedestrian groups, we assumed that the trajectories relating groups are already identified. In our study, this identification is performed by human coders. Nevertheless, it can also be done by a group recognition algorithm. Obviously, recognizing whether two (or more) pedestrians are or are not part of a group is an interesting and not trivial problem. Concerning this issue, we refer the reader to [23], which leads to an accuracy of over 90% with similar experimental conditions, pedestrian profile and sensory information. In [23], the experiments are carried out in public spaces with a low density, the subjects are uninstructed pedestrians and the analysis uses range data with similar accuracy. Therefore, we believe the reported group detection accuracy of [23] will apply to the current data set as well.

The data set was labeled by a human coder (*primary coder)*, based on video and trajectory information with respect to several intrinsic group features. One specific feature refers to the apparent social relation, where the possible options are colleagues, family, couple or friends. These correspond to the domains of coalitional, attachment, mating and reciprocal, respectively, as defined by Bugental [42].

In order to test the reliability of this coding process, two other human coders (*secondary coders*) were asked to label a portion of the entire data. This portion is chosen arbitrarily and is the same for the two secondary coders (This means that the inter-rater agreement analysis is carried out based on the labels of all three coders on the same subset of the data.). The inter-rater reliability of this labeling process is evaluated using several prominent statistical measures [1], all of which indicate that the coders are in considerable agreement [48]. As a result of this labeling process, the number of observations from each relation is determined to be as in Table 1.

**Table 1:** Number of observations.

Social relation	Relation category [42]	# of observations
Colleagues	Coalitional	359
Families	Attachment	238
Couples	Mating	100
Friends	Reciprocal	322

Although [1] establishes that this coding process attains satisfactory inter-agreement rates, for the purpose of this paper it is beneficial to take a closer look at the correspondence of coders’ labels. Namely, the correspondence presented in Table 2 reveals that certain social relation categories are easier to confuse. This may be due to the fact that some categories are inherently harder to identify for humans, but it may also be related to coders’ notion of the concepts. Specifically, families and couples have a relatively high rate of confusion. The existence of a bond of marriage could be the main determining factor in distinguishing families from couples in mixed gender dyads, which may depend on the individual coder’s values of judgment (such as marriage age, see also the discussion in Families with children section). In addition, although colleagues attain a very good rate of agreement, there is a relatively high rate of confusion between friends and colleagues. This is probably due to the fact that although visual cues such as clothing (e.g. suit or uniform) provide a convincing evidence regarding professional relation, such visual evidence implicating friendship is to a more limited extent.

**Table 2:** Average agreement between the primary coder and the two secondary coders. The entry (*i*, *j*) corresponds to the rate with which a dyad in category *i* according to the primary coder was labeled as *j* by the secondary coders.

	Colleagues	Families	Couples	Friends
Colleagues	92%	1%	1%	6%
Families	6%	73%	12%	9%
Couples	0%	9%	84%	7%
Friends	10%	11%	3%	76%

### Observables and empirical distributions

Zanlungo et al. show that social relation strongly affects the pdf of group velocity, distance between members, and projection of distance along and orthogonal to the direction of motion [1]. From the results of [1], it is clear that the proposed method could also be used to infer other “intrinsic properties” of groups, such as the gender of its members. Based on these results (and on our previous works on group recognition), this study proposes a recognition method based on empirical pdfs of relevant distributions. It would thus seem natural to use the same observables of [1] in our work.

However, [1] was based on the theoretical work of [7], that analyses groups under the assumption that their relative velocity is negligible with respect to the group velocity and thus did not perform an analysis of relative velocity. Indeed, it can be seen from the figures in Empirical observations section that velocity difference is of an order of magnitude smaller than group velocity. Nevertheless, we know from our previous work on social interaction of groups [2] that the distribution of this observable is affected by the nature of interaction (i.e. gestures), and thus decided to include it in our analysis. Furthermore, for recognition of group relation, the height of its members (as provided by the tracking algorithm) is of help (since height difference between couples or family members is, from a probabilistic viewpoint, more pronounced than height difference between colleagues and friends. Refer to [49] and the figures in Empirical observations.).

For these reasons, we decided to include in our analysis also velocity difference and height difference. Nevertheless, in order not to extend too much the dimension of observable space, we decided to use only one of the relative distance observables, namely absolute distance (i.e. interpersonal-distance).

Although in this work we use a supervised learning approach over predefined observables, theoretically the same task could be handled using unsupervised machine learning methods such as DNN directly on group trajectories. This latter approach may be more effective in the future. However, at the current stage, given the limited amount of available data and the high number of dimensions of the variable (i.e. feature) space, a DNN based method would nevertheless need some informed choice of observables. We thus believe that the first step that we are performing in this work may be a guiding light also for any future work based on DNN or similar unsupervised machine learning methods.

We now proceed to a formal definition of the observables.

#### Observables

**Fig 1:**
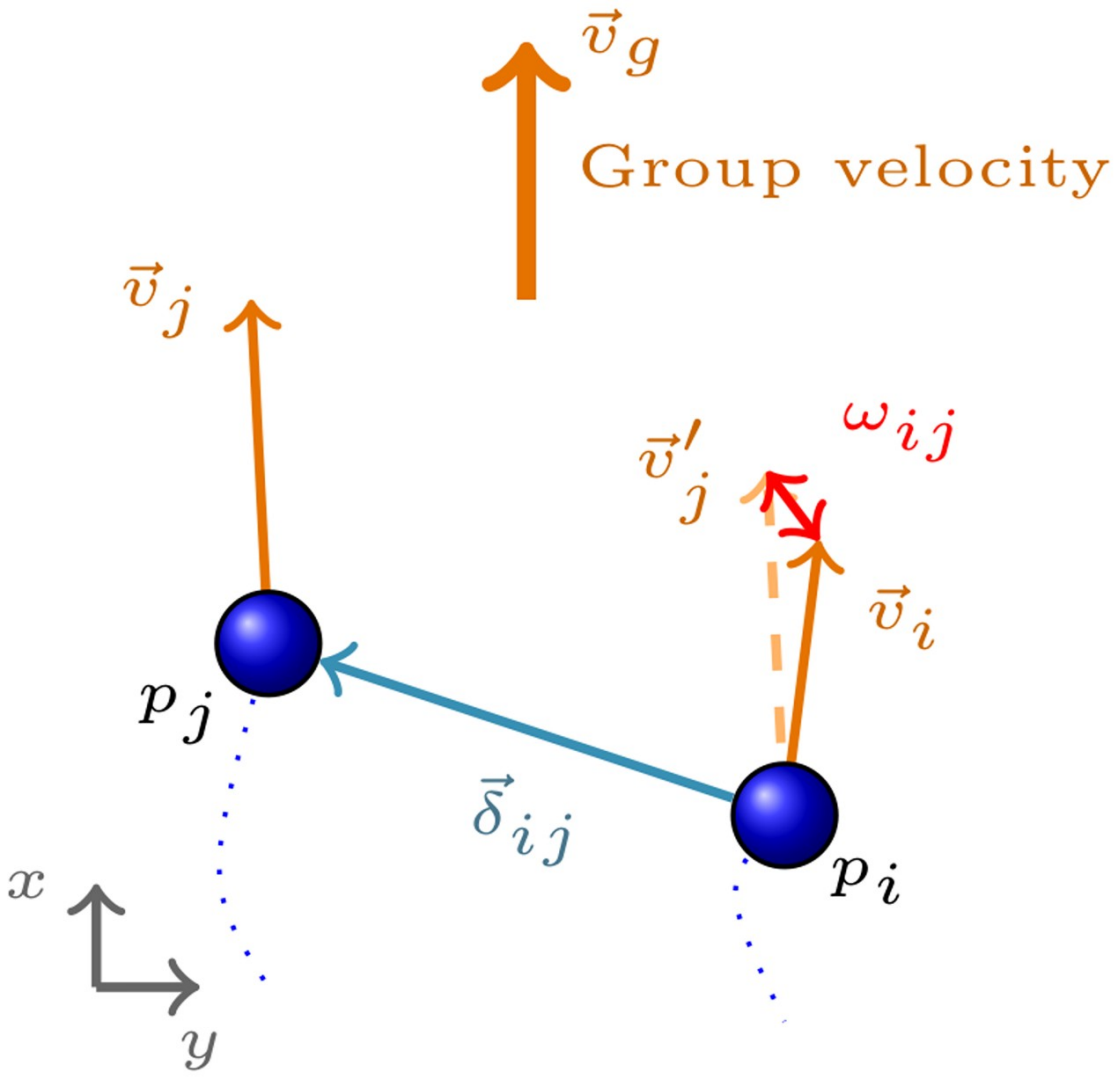
The observables depicted on a sample dyad {*p*_*i*_, *p*_*j*_}.

In examining the joint behavior, we focus on the following observables: inter-personal distance, velocity difference of the peers, group velocity, and height difference. In what follows, we provide the definitions of these observables on a sample dyad {*p*_*i*_, *p*_*j*_} depicted in Fig 1.

1. *Interpersonal distance*, *δ*_*ij*_, is defined as the magnitude of the relative distance vector between the peers. If the position of pedestrian *p*_*i*_ is given on an arbitrary frame of reference by ${\vec{x}}_{i}=\left(x_{i},y_{i}\right)$, we have
  $$\begin{array}{c} \delta_{ij}=\left\|{\vec{\delta }}_{i,j}\right\|=\sqrt{{\left(x_{i}-x_{j}\right)}^{2}+{\left(y_{i}-y_{j}\right)}^{2}}. \end{array}$$
2. *Group velocity magnitude* (to which we will often refer as simply “group velocity”) of the dyad {*p*_*i*_, *p*_*j*_}, $v_{i,j}^{g}$, is the magnitude of the average instantaneous velocity of the peers,
  $$\begin{array}{c} v_{i,j}^{g}=\|\frac{{\vec{v}}_{i}+{\vec{v}}_{j}}{2}\|. \end{array}$$
3. *Relative velocity magnitude*, *ω*_*ij*_, is defined as the magnitude of the difference vector,
  $$\begin{array}{c} \omega_{ij}=\left\|\vec{v_{i}}-\vec{v_{j}}\right\|. \end{array}$$
4. *Height difference* between peers is denoted by *η*_*ij*_,
  $$\begin{array}{c} \eta_{ij}=|\eta_{i}-\eta_{j}|, \end{array}$$
  where *η*_*i*_ and *η*_*j*_ stand for the height of the pedestrians *p*_*i*_ and *p*_*j*_, respectively.

Note that these observables represent motion dynamics relating two pedestrians and, thus, they can be directly computed in case of dyads, whereas a nontrivial adjustment would be necessary to define them for larger groups. Particularly, it would be necessary to handle pairwise relations and the hierarchy and transitivity between subgroups or group members, who are not immediate neighbors [50].

#### Empirical observations

This section presents the pdfs of the four observables integrated over all time points and all dyads throughout the observed period. Also, in each figure we present four curves, each corresponding to a different kind of social relation.

**Fig 2:**
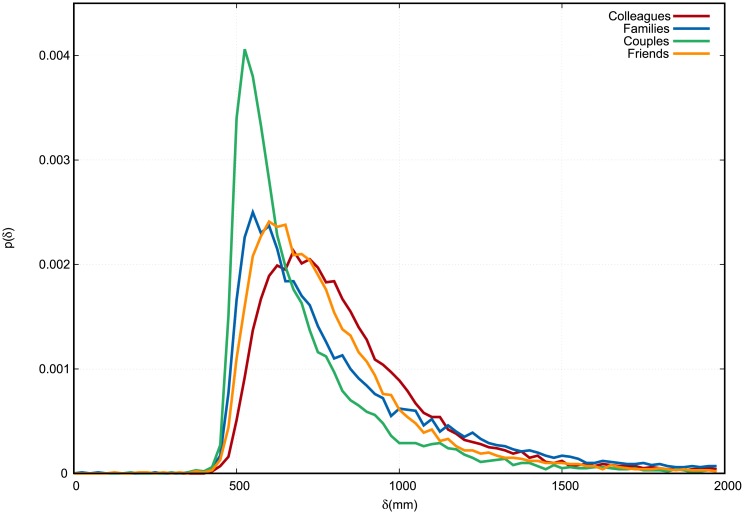
Empirical distribution of interpersonal distance *δ*. Colleagues in red, families in blue, couples in green, friends in orange. The continuous curves connect the discrete values of each histogram bin, which are normalized in such a way that the area under the curve equals 1. Distances are measured in millimeters.

The inter-personal distance pdfs regarding the entire set of social relations, i.e. colleagues, families, couples and friends, are presented in Fig 2. It may be observed that peaks of the distributions are assumed at different values of *δ*, and more in detail in an ascending order for couples, families, friends and colleagues. On the other hand, families and colleagues present fatter tails for large *δ* values than couples and friends.

**Fig 3:**
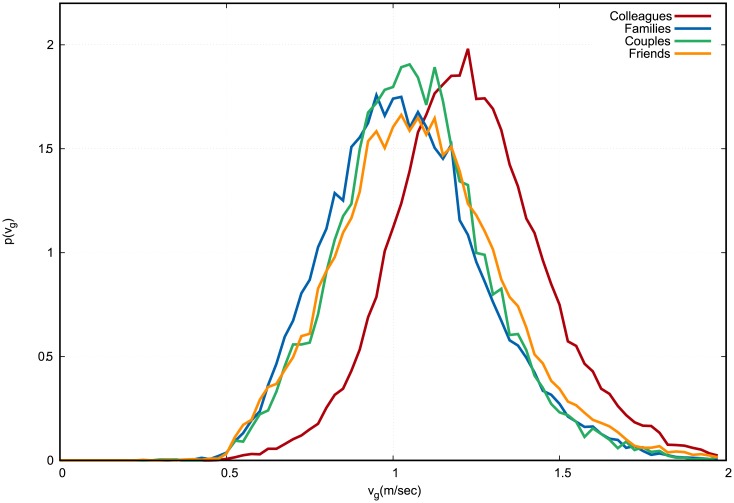
Empirical distribution of group velocity *v*^*g*^. Colleagues in red, families in blue, couples in green, friends in orange. The continuous curves connect the discrete values of each histogram bin, which are normalized in such a way that the area under the curve equals 1. Velocities are measured in meters per second.

The support of the distribution of group velocity *v*^*g*^ is displaced to higher values for colleagues, while it is very similar for the remaining social relations (see Fig 3).

**Fig 4:**
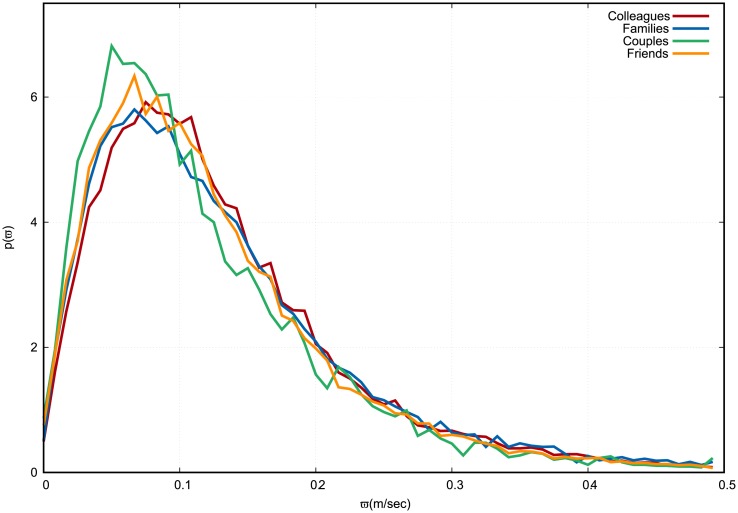
Empirical distribution of relative velocity magnitude *ω*. Colleagues in red, families in blue, couples in green, friends in orange. The continuous curves connect the discrete values of each histogram bin, which are normalized in such a way that the area under the curve equals 1. Velocities are measured in meters per second.

The peak of the *ω* distribution assumes a lower value for couples and a higher value for colleagues (see Fig 4) and it is very similar for families and friends.

Concerning the last observable of interest, namely, height difference *η*, we notice that it assumes clearly a lower peak and fatter tails for families and couples, as compared to colleagues and friends.

**Fig 5:**
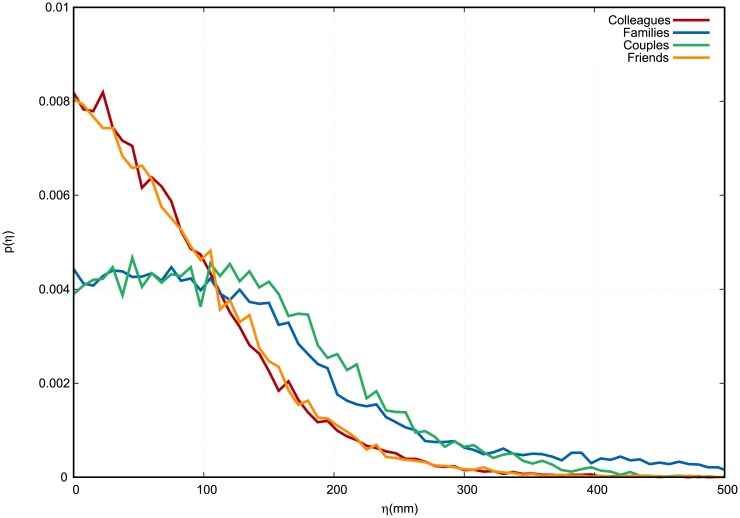
Empirical distribution of height difference of peers *η*. Colleagues in red, families in blue, couples in green, friends in orange. The continuous curves connect the discrete values of each histogram bin, which are normalized in such a way that the area under the curve equals 1. Distances are measured in millimeters.

From Figs 2–5, it is evident that the distribution of *ω* is the one to be least affected by social relation. Nevertheless, a standard Analysis of variance (ANOVA), in which *p*-values are all found to be smaller than 10^−4^, shows that social relation has a statistically significant effect on all observables including *ω*.

#### Hierarchical vs Non-hierarchical recognition

In [1], an explicit annotation of the “purpose of visit” of pedestrian groups to the experiment venue was performed. Namely, coders were asked to identify “work-oriented” and “leisure-oriented” groups. Not surprisingly, the former coincided almost perfectly with colleagues, while the latter with families, couples and friends. It is thus convenient to identify the union of these latter relations as “leisure-oriented”. The observable pdfs for the leisure-oriented groups are then just a weighted sum of the distributions relating families, couples, and friends, and they are compared to the distributions of colleagues in S1 Appendix. Leisure actually represents a *purpose* and not a social relation. Nevertheless, based on the labels of the data set used by [1], it is reasonable to identify “leisure-oriented” as the union of the social relations of family, couple, and friends, and thus we refer to it as a *complementary relation*.

The distinction of the observable pdfs relating “work-oriented” and “leisure-oriented” groups is very clear (in particular for *v*^*g*^), and thus it appears reasonable that the automatic recognition of these two (meta) categories (i.e. work-oriented and leisure-oriented) should be easier than the full recognition of the four relational categories (i.e. colleagues, families, couples and friends).

We both perform a “hierarchical” recognition, where we assess initially the ability of our method to recognize dyads as belonging to “work-oriented” or “leisure-oriented” categories (stage-1), and subsequently its ability to distinguish between the leisure-oriented subcategories (i.e. families, couples, and friends) when only these are presented to the algorithm (stage-2). Furthermore, we also provide the results of a one-step (non-hierarchical) recognition process, in which dyads are directly recognized as belonging to social relation categories of colleagues, families, couples or friends.

#### Families with children

In the analysis of the results of [1], it was realized that the choice of the Japanese language words used to define the social relation had probably made implications on the age of the pedestrians. Namely, the word *koibito*, used to identify the couple relation, is often used to refer to “young” couples, and as a result older couples were often (but not always) identified as belonging to the family relation.

A possible way to avoid this ambiguity is to take advantage of the age labeling in the data set, and remove from the trajectory set those dyads, in which both peers are older than 15 years (i.e. using only *families with children*). Therefore, it should be noted that this is not a *further preprocessing* step, neither is it an *improvement* of the data set labeling. Imposing the aforementioned condition (on age) on all dyads labeled as family (see Table 1), the number of samples decreases from 238 to 57, and the corresponding observable pdfs are found to be as shown in S6 Appendix.

### Recognition of social relations

We contrast using two different approaches for the recognition of social relation, while handling the temporal information encapsulated in the data. Before describing our recognition method(s), we would like to distinguish between these two very different ways (to use empirical observable pdfs to recognize social relation of dyads). Namely, we consider the following two approaches:

- **Event-based**: Such methods compare a single observation of the group (i.e. its state at a given time instant in terms of the four observables) and evaluate, through the empirical pdfs and Bayes theorem, the probability that such observation belongs to a given relational category. Such methods are thus able to *provide a probability for the group belonging to each relational category at each time instant*.
- **Trajectory-based**: Such methods observe the group over a time window and build its particular observable pdfs. The obtained pdfs are then compared to the empirical pdfs of each social relation category and the difference between pdfs is evaluated using some kind of functional metric. Such methods are thus able to *indicate, after a complete observation over a time span, which social relation category presents pdfs more similar to the behavior of the group in question*.

Since we opt for a Bayesian approach for the event-based recognition, henceforth we refer to it as “Bayesian method”, whereas the trajectory-based recognition is termed as “Functional metric method”, since it relies on comparison of two distributions through a metric.

Theoretically, the Bayesian method seems to be more powerful, since it can be applied even on a single observation and can also be applied to full trajectories, (e.g. by averaging the corresponding probabilities over the observation duration), and provide an answer that has a well-defined probabilistic interpretation. This does not nevertheless mean that it is better than functional metric methods from a practical point of view (i.e. provide better recognition rates), and for this reason, both approaches are tested in our work.

#### Bayesian method

In this section, we describe the event-based approach that we propose for estimating the social relation. As briefly mentioned in Recognition of social relations section, in doing that, we take a Bayesian stand-point and compute the conditional probability that a given set of observations comes from a group engaged in a particular social relation.

Suppose that from a pair of pedestrians {*p*_*i*_, *p*_*j*_}, at time *t* (global time as recorded by the tracking system) we gather an observation vector denoted by S(t). In the rest of the analysis, we drop the indices (*i*, *j*) for the simplicity of the notation and *t* is omitted, where it is not necessary. This vector is composed of the observables of interpersonal distance, group velocity, relative velocity magnitude, and height difference between the peers, i.e. $S=[\delta ,v_{g},\omega ,\eta {]}^{T}$.

Let us denote social relation by *r*, where *r* is either colleagues, families, couples or friends. We compute the probability that the observation vector S, observed at time *t*, comes from a group in social relation of *r*, $P_{t}\left(r|S\right)$, as follows,

$$\begin{array}{c} P_{t}\left(r|S\right)=\frac{P_{t}\left(S|r\right)P_{t}\left(r\right)}{P_{t}\left(S\right)}. \end{array}$$ (1)

Here, $P_{t}\left(r|S\right)$ is the posterior probability that a group belongs to a particular relation *r* given the observation vector S. In addition, $P_{t}\left(S|r\right)$ is the likelihood term and *P*_*t*_(*r*) is the prior probability of social relation.

Although in Empirical observations section, we analyzed 1-D pdfs, the observation vector S takes values in a subset of the 4-D vector space $R^{4}$. The pdfs shown in Empirical observations section are thus obtained by showing the dependence on a single variable and integrating on the remaining three.

From a theoretical standpoint, there is no difficulty in operating directly with 4-D pdfs in Eq 1, but from a practical point of view this results to be very difficult.

Namely, empirical pdfs $P_{t}\left(S|r\right)$ are obtained by dividing the relevant $R^{4}$ subset in discrete bins, and then by evaluating the probability of each bin as the ratio of the number of data points falling in it to the total number of data points. The size of the bin is crucial, since it has to be small enough to be sensible to variations in pdfs between different relations *r*, but at the same time it has to be large enough to contain a sufficient (i.e. representative) number of data points. This phenomenon is known commonly as “the Curse of Dimensionality”. In order to cope with this issue, we roughly want to have, for all bins *i* corresponding to the bulk of the distribution,

$$\begin{array}{c} 1/\sqrt{N_{i}}\ll 1, \end{array}$$

where *N*_*i*_ is the number of observations in bin *i*, since fluctuations are of order $1/\sqrt{N}$. Since maxima of the pdfs are displaced usually by an order of a few hundredths of the range extension assumed by the variable domain (refer to figures in Empirical observations section), we need to have roughly an order of 10^2^ bins in each dimension.

For 1-D variables, this does not represent a problem, since our empirical observation set contains an order of 10^4^ data points and thus fluctuations are expected to be relatively small. But if we operate in the full 4-D space, we would end up having ≈ 10^8^ bins, and our observation set is too small to calibrate 4-D empirical observables. In agreement with this dimensional analysis, the difference between the size of the discrete space and observational set is so large that also attempts to “smooth” the 4-D pdfs were fruitless.

A solution to this problem consists in assuming that the observables *δ*, *v*^*g*^, *ω*, and *η* are conditionally independent (the validity of this assumption is verified by evaluating the normalized entropy distance in S2 Appendix).

This enables expressing the likelihood term with the following product,

$$\begin{array}{c} P_{t}\left(S|r\right)=P_{t}\left(\delta |r\right)P_{t}\left(v_{g}|r\right)P_{t}\left(\omega |r\right)P_{t}\left(\eta |r\right). \end{array}$$ (2)

For each conditional probability in Eq 2, we use the empirical distributions, built according to the process of discretization in bins described above.

As for an initial value for our prior belief, *P*_0_(*r*), we adopt an equal probability to avoid any bias. Since we have four possible categories of social relation, this leads to the following,

$$\begin{array}{c} P_{0}\left(r\right)=(\begin{array}{cccc} 0.25 & 0.25 & 0.25 & 0.25 \end{array}). \end{array}$$ (3)

The marginal probability term appearing in the denominator of Eq 1, $P_{t}\left(S\right)$, i.e. the probability of observing S regardless of the relation *r*, does not need to be computed explicitly, since it is independent of *r*, and thus can be obtained by requiring normalization of the probabilities. Indeed, from a computational standpoint, we define

$$\begin{array}{c} {\tilde{P}}_{t}\left(r|S\right)=P_{t}\left(S|r\right)P_{t}\left(r\right), \end{array}$$ (4)

and compute

$$\begin{array}{c} P_{t}\left(r|S\right)=\frac{{\tilde{P}}_{t}\left(r|S\right)}{\sum_{r^{\prime }}{\tilde{P}}_{t}\left(r^{\prime }|S\right)}. \end{array}$$ (5)

As time elapses, we propose updating the prior as in the following equation,

$$\begin{array}{c} P_{t}\left(r\right)=\left(1-\alpha \right)P_{0}\left(r\right)+\alpha P_{t-1}\left(r|S\right), \end{array}$$ (6)

where the parameter *α* defines the rate of update and can assume values between 0 and 1 [51]. Specifically, *α* = 0 corresponds to not updating the priors (i.e. using always the initial guess in Eq 3), while *α* = 1 corresponds to using the previous result as a prior and *α* = 0.5 corresponds to using as a prior the average between the initial guess and the previous result. Although any *α* ∈ [0, 1] is admissible, in our analysis we will limit ourselves to the three cases above, namely *α* = {0, 0.5, 1}.

We comment on the special case in which P(S|r)=0. This situation has actually to be analyzed corresponding to two different cases, namely (i) when at least one of the *P* is nonzero, ∃r:P(S|r)≠0, and (ii) when they are all equal to zero P(S|r)=0∀r. The first case can be handled without problems by our method, and yields $P_{t}\left(r|S\right)=0$. This happens because we are observing a completely acceptable S value that has never been observed before in that particular social relation category *r*. Here, by “acceptable”, we mean that it is observed in our data set for a different social relation. According to our probabilistic method, this leads to having $P_{t}\left(r|S\right)=0$, i.e. resulting in the conclusion that the group cannot possibly belong to the category *r*. If *α* ≠ 1, a nonzero value of P(r|S) can still be attained in the future (see Eq 6), while for *α* = 1 the prior (and thus the conditional probability) for the category *r* is assigned to zero for the remainder of the trajectory.

The case P(S|r)=0∀r is conceptually different. This corresponds to a value that has never been observed before in any category or to a value that has been discarded as a “proper group behavior” observation by putting it beyond the domain range of empirical pdfs. Regarding properness of group behavior, both from a theoretical and a practical standpoint, it is necessary to limit the $R^{4}$ subset in which we compute empirical observables. The practical reason is to keep a computationally reasonable number of bins in our discrete pdfs. But also theoretically, it is important to assume, as proper group behavior, the range of observables in which the bulk of the pdf is present. For example, consider the distribution of *δ*, which goes quickly to zero above 2 m (see Fig 2). Individual pedestrians in a social group can have larger distances (e.g. after splitting), but it is fair to admit that at such a distance, they are not behaving as a group. For this reason, we limit the range of the empirical *δ* pdf at 2 m. Similarly, following the discussion of [7], to consider only proper walking group behavior, we filter out group velocities below 0.5 m/s. This threshold is chosen since, as discussed in [52], it allows to separate the Gaussian distribution that characterizes walking pedestrians from the lower-value Rayleigh distribution characterizing standing behavior.

Here, it is noteworthy to mention that similar to *δ* and *v*^*g*^, also *ω* and *η* are considered to belong to certain reasonable ranges. This anticipation is beneficial in identifying nonsensical measurements (i.e. potential mis-measurements). In addition, we also choose the number of bins for computing the histograms of the observable values falling between their respective ranges. In this manner, we determine the resolution of the empirical distributions. This pair of choices (i.e. range and number of bins) helps in building empirical distributions representing the relevant part of the observable space at a proper level of granularity (i.e. with sufficiently large but also affordable number of bins). The lower bound, upper bound and number of bins for all observables are taken as presented in Table 3.

**Table 3:** Hyper-parameters used in building empirical distributions.

	Lower bound	Upper bound	Number of bins
*δ*	0 m	2 m	80
*v*^*g*^	0.5 m/s	2 m/s	90
*ω*	0 m/s	0.5 m/s	60
*η*	0 m	1.125 m	150

When observables fall outside their permissible range, Eq 5 is undefined, since the denominator assumes value 0. In this situation, the method is telling us that the pedestrians are not in any relational category *r*. Since we assume that only proper groups in one of the categories are passed to our method, in this case we assume *zero knowledge* and set $P_{t}\left(r|S\right)=1/4\;\forall r$.

#### Functional metric methods

The trajectory-based methods rely on a comparison between the empirical pdfs P(S|r) derived as described in Bayesian method section and the dyad pdf $P_{d}\left(S\right)$, which is built following the same discretization process as for P(S|r), i.e. using all the S values corresponding to the observed trajectory of the dyad. In case of actual practical applications, the trajectories could be observed during a fixed time span *T*, at the end of which the pdf $P_{d}\left(S\right)$ could be built and the dyad relation evaluated.

If a proper functional metric *M*(*f*_1_, *f*_2_) [53] is defined for *f*_1,2_ belonging to the relevant space of probability density functions, then we may evaluate the distance between the pdf of the dyad and the empirical pdfs of different social relations, as

$$\begin{array}{c} D\left(d,r\right)=M(P_{d}(S),P\left(S|r\right)), \end{array}$$ (7)

and assign to the dyad the relation with the smallest distance. Since the functional metric provides a continuous value, one could try to use them to define, again in a continuous way, a “probability” for each relation. We are not anyway aware of a rigorous method to perform the conversion between the metrics and probabilities, and thus in the remainder of this work, the trajectory-based methods are assumed to provide a purely discrete assignment of social relation to dyads,

$$\begin{array}{c} r_{d}=\underset{r}{\mathrm{argmin}}(D\left(d,r\right)). \end{array}$$ (8)

Also for these methods we face the problem of building fully 4-D pdfs $P_{d}\left(S\right)$, and thus these pdfs and the corresponding metric computations are derived under the assumption of independence, according to the procedure described below for the proposed metric.

The Earth Mover’s Distance (EMD) between two distributions is proportional to the minimum amount of *work*, which is required to morph one distribution into another. If the two distributions are of *equal weight* (i.e. if they have the same integral, as in case of pdfs), mathematically speaking, it defines a true metric, and is also termed as the Wasserstein metric [54].

The name “Earth Mover’s Distance” derives by visualizing each distribution as a “pile of dirt”, and the functional distance between the two distributions as the minimum effort or work, defined as the amount of dirt multiplied by traveled distance, which is required to morph one into the other. The relevance of the EMD to the problem of comparing pdfs may be understood examining the case of histograms *P* and *Q* with just a single occupied bin (“*δ* functions”)

$$\begin{array}{c} P\left(i\right)=\delta_{i,j},\;\;\;\;\;\;Q\left(i\right)=\delta_{i,k}. \end{array}$$

If the two distributions are compared with a *l*_2_ metric [53] the result does not depend on the distance between the bins

$$\begin{array}{c} l_{2}\left(P,Q\right)=\sqrt{\sum_{i}{\left|P\left(i\right)-Q\left(i\right)\right|}^{2}}=\sqrt{2}, \end{array}$$ (9)

while in the EMD case it results to be proportional to the distance |*j* − *k*| (a property that makes EMD particularly relevant to the problem of comparison between pdfs [55]). A formal definition of EMD is reported in S3 Appendix.

However, concerning our specific application, there is a high computational cost to compare two full dimensional distributions in terms of EMD. In such cases, often approximation methods are employed [56]. In our case, rather than approximating the EMD, we prefer exploiting the fact that the observable distributions are shown to be conditionally independent and this may help in deriving an upper bound of EMD based on its values concerning each dimension of the observable space. In S3 Appendix, we show that an upper bound for the EMD between two probability density functions concerning a multivariate random variable with independent components can be provided by the sum of the EMD along each dimension. We therefore use this upper bound of the EMD as our functional metric used to calculate *D*(*d*, *i*) in Eq 7.

In S4 Appendix, we compare to two other methods to measure the difference between probability density functions, namely the Kullback-Leiber and Jensen-Shannon divergences. According to our results, the EMD-based method largely outperforms the methods based on the Kullback-Leiber and Jensen-Shannon divergences.

### Assessment of performance

While the functional metric methods just assign a given relation to each dyad, and thus their performance is quite easy to evaluate (basically by computing a confusion matrix), the Bayesian method provides us a probabilistic answer at each observation instant *t*, and thus its performance can be assessed in different ways. Namely, we can assess the performance of the Bayesian method by comparing the proposed relation assignment with the ground truth at each event (evaluation by-event) or at the end of each trajectory (evaluation by-trajectory). Obviously, only the evaluation by-trajectory allows for a straightforward comparison to the functional metric methods. Nevertheless, to evaluate the Bayesian method by-trajectory, we have to properly define how the event-based information is coded at the trajectory level.

#### Assessment of the Bayesian method

In the data set, a “ground truth” relation $r_{d}^{GT}$ is assigned to each dyad, *d*, as explained in Data set section and $r_{d}^{GT}\left(t\right)=r_{d}^{GT}$. Additionally, for each observation vector S, the Bayesian method provides a probability that the dyad belongs to a given social relation *r*, as $P_{t}\left(r|S\right)$.

We may define *r*_*d*_(*t*) as the social relation, to which the maximum probability is assigned for dyad *d* at time *t*, namely

$$\begin{array}{c} r_{d}\left(t\right)=\underset{r}{\mathrm{argmax}}(P_{t}\left(r|S\right)), \end{array}$$ (10)

(in case the maximum value appears multiple times, we randomly pick one occurrence).

Let us now define *R*_*d*,*i*_ as the set of times *t* at which dyad *d* satisfies *r*_*d*_(*t*) = *i*, and *R*_*i*_ as the set including all dyads *d* and times *t* such that *r*_*d*_(*t*) = *i*, namely

$$\begin{array}{c} R_{i}=\underset{d}{⋃}R_{d,i}. \end{array}$$ (11)

Furthermore, we define $R_{i}^{GT}$ as the set including all dyads *d* and times *t* such that $r_{d}^{GT}\left(t\right)=i$. By denoting the cardinality of a set *A* as |*A*|, we may now define the confusion matrix,

$$\begin{array}{c} C_{ij}^{be}=\frac{|R_{j}⋂R_{i}^{GT}|}{|R_{i}^{GT}|}, \end{array}$$ (12)

where *i* and *j* are any two social relation categories. We call this assessment method “binary-by-event”.

In addition to this assessment based on events, we consider another approach based on trajectories, i.e. assigning a single category (i.e. social relation) to each trajectory (i.e. each dyad). To that end, we first define $r_{d}^{v}$ as the social relation that gets “more votes” along the entire trajectory,

$$\begin{array}{c} r_{d}^{v}=\underset{i}{\mathrm{argmax}}\left|R_{d,i}\right|. \end{array}$$ (13)

We then define $R_{i}^{v}$ as the set of all dyads *d* with $r_{d}^{v}=i$, and ${\tilde{R}}_{i}^{GT}$ as the set including all dyads *d* such that $r_{d}^{GT}=i$. Finally, we define

$$\begin{array}{c} C_{ij}^{v}=\frac{|R_{j}^{v}⋂{\tilde{R}}_{i}^{GT}|}{|{\tilde{R}}_{i}^{GT}|}. \end{array}$$ (14)

We call this assessment method “binary-by-trajectory-voting”.

The most important reason for proposing the latter approach is to provide a common ground of comparison between the Bayesian and functional metric methods. In other words, since functional metric methods yield an outcome for every trajectory rather than every event, a direct comparison between the outcomes of Eq 12 and the functional metric methods is not possible. Converting our event-based probabilities into trajectory level as in Eq 13 provides a basis for a fair comparison of the two methods.

#### Assessment of functional metric methods

Functional metric methods treat the entire trajectory as a single entity and yield a single decision for every dyad.

Let us assume that the proposed method chooses the social relation category *r*_*d*_ (see Eq 8), which is characterized by having the pdf with lowest distance from the observed one, as the *recognized* relation. We may now define ${\tilde{R}}_{i}$ as the set of dyads *d* such that *r*_*d*_ = *i*, and the confusion matrix *C* as

$$\begin{array}{c} C_{ij}=\frac{|{\tilde{R}}_{j}⋂{\tilde{R}}_{i}^{GT}|}{|{\tilde{R}}_{i}^{GT}|}. \end{array}$$ (15)

Clearly, *C*_*ii*_ gives us the rate of correct recognition for social relation *i*.

#### Confusion matrices for different steps of hierarchical methods

To describe the results of the stage-1 of the hierarchical decision method, we will show two different confusion matrices, namely a 2 × 2 one, in which both true and assigned classes assume either “work-oriented” or “leisure-oriented”, and a 4 × 2 one, in which the true class assumes either colleagues, families, couples or friends. In the 4 × 2 confusion matrix, the assigned class assumes “work-oriented” or “leisure-oriented” as in case of the 2 × 2 case. Stage-2 will be assessed by a 3×3 matrix, in which both true and assigned classes assume either families, couples or friends.

## Results

From a practical viewpoint, empirical pdfs P(S|r) should be built using all available data. Nevertheless, while evaluating the method, we need to divide our data into training and testing sets. Obviously, only the training set is used to build the empirical pdfs. We randomly select 30% of dyads as a training set, and use the remaining 70% to test our methods. Moreover, repeating this procedure 50 times, we compute the mean performance values. By randomly picking 30% of the entire sample and repeating this procedure 50 times, the probability that a particular sample is not used in training is below 10^−3^ (results obtained using training sets corresponding to 15% and 50% of the entire data set are reported in S7 Appendix). Concerning the Bayesian method, we show -in general- the results only for *α* = 1 using the “by-event” assessment (Eq 12), while the results obtained for different values of *α* are discussed in Results of Bayesian approach for *α* ≠ 1 and shown in detail in S5 Appendix, and the “by-trajectory” results are shown for the non-hierarchical process in Comparing Bayesian approach and functional metric methods section. In Tables 4–19, GT stands for ground truth class.

**Table 4:** Binary-by-event $C_{ij}^{be}$, hierarchical stage-1, *α* = 1 (in %).

		Work	Leisure
GT	Work	**73.69**	26.31
	Leisure	29.18	**70.82**

**Table 5:** Binary-by-event $C_{ij}^{be}$, hierarchical stage-1, *α* = 1 (in %) with detailed confusion rates.

		Work	Leisure
GT	Colleagues	**73.69**	26.31
	Families	22.19	**77.81**
	Couples	15.58	**84.42**
	Friends	39.62	**60.38**

**Table 6:** Earth mover’s distance *C*_*ij*_, hierarchical stage-1 (in %).

		Work	Leisure
GT	Work	**81.43**	18.57
	Leisure	37.80	**62.20**

**Table 7:** Earth mover’s distance *C*_*ij*_, hierarchical stage-1 (in %) with detailed confusion rates.

		Work	Leisure
GT	Colleagues	**81.43**	18.57
	Families	30.12	**69.88**
	Couples	27.66	**73.34**
	Friends	46.65	**53.35**

**Table 8:** Binary-by-event $C_{ij}^{be}$, hierarchical stage-2, *α* = 1 (in %).

		Families	Couples	Friends
GT	Families	**41.52**	23.94	34.54
	Couples	32.14	**38.17**	29.69
	Friends	18.43	14.53	**67.04**

**Table 9:** Earth mover’s distance *C*_*ij*_, hierarchical stage-2 (in %).

		Families	Couples	Friends
GT	Families	32.02	**37.63**	30.35
	Couples	12.91	**60.57**	26.51
	Friends	8.43	26.77	**64.80**

**Table 10:** Binary-by-event $C_{ij}^{be}$, non-hierarchical, *α* = 1 (in %).

		Colleagues	Families	Couples	Friends
GT	Colleagues	**68.31**	7.29	5.37	19.03
	Families	18.10	**38.92**	20.66	22.32
	Couples	13.58	31.11	**36.57**	18.75
	Friends	34.19	16.66	12.74	**36.41**

**Table 11:** Earth mover’s distance *C*_*ij*_, non-hierarchical (in %).

		Colleagues	Families	Couples	Friends
GT	Colleagues	**71.63**	2.88	8.90	16.60
	Families	22.23	24.07	**35.22**	18.48
	Couples	17.80	11.97	**56.14**	14.09
	Friends	**35.62**	5.49	26.28	32.60

**Table 12:** Binary-by-trajectory-voting $C_{ij}^{v}$, non-hierarchical, *α* = 1 (in %).

		Colleagues	Families	Couples	Friends
GT	Colleagues	**73.18**	5.93	4.59	16.30
	Families	19.02	**41.01**	19.40	20.57
	Couples	15.20	26.69	**39.89**	18.23
	Friends	**39.88**	13.84	12.66	33.63

**Table 13:** Binary-by-trajectory-voting $C_{ij}^{v}$, non-hierarchical, *α* = 1 (in %), for trajectories over median length.

		Colleagues	Families	Couples	Friends
GT	Colleagues	**63.49**	8.54	4.14	23.84
	Families	16.81	**35.39**	20.41	27.39
	Couples	16.41	30.92	**32.10**	20.56
	Friends	29.24	20.76	9.73	**40.27**

**Table 14:** Earth mover’s distance *C*_*ij*_, non-hierarchical (in %), for trajectories over median length.

		Colleagues	Families	Couples	Friends
GT	Colleagues	**67.73**	2.49	9.76	19.78
	Families	16.46	22.09	**38.55**	22.90
	Couples	18.15	11.49	**56.36**	14.00
	Friends	**32.37**	12.27	23.67	31.69

**Table 15:** Binary-by-event $C_{ij}^{be}$, non-hierarchical, *α* = 0.5 (in %).

		Colleagues	Families	Couples	Friends
GT	Colleagues	**65.78**	7.55	11.46	15.21
	Families	20.12	29.11	**31.11**	19.65
	Couples	18.71	17.34	**47.75**	16.20
	Friends	**37.67**	13.07	21.35	27.91

**Table 16:** Binary-by-event $C_{ij}^{be}$, non-hierarchical, *α* = 0 (in %).

		Colleagues	Families	Couples	Friends
GT	Colleagues	**59.67**	8.42	13.80	18.11
	Families	20.46	27.58	**30.69**	21.26
	Couples	19.27	17.75	**45.25**	17.72
	Friends	**36.22**	13.23	22.05	28.50

**Table 17:** Binary-by-trajectory-voting $C_{ij}^{be}$, non-hierarchical, *α* = 0, using prior rates of representation as initial priors.

		Colleagues	Families	Couples	Friends
GT	Colleagues	**70.98**	7.53	1.03	20.47
	Families	31.18	30.99	4.19	**33.64**
	Couples	30.79	28.02	8.32	**32.88**
	Friends	**46.00**	13.45	1.90	38.65

**Table 18:** Binary-by-event $C_{ij}^{be}$, non-hierarchical, families with children, *α* = 1 (in %).

		Colleagues	Families	Couples	Friends
GT	Colleagues	**69.12**	4.61	6.21	20.07
	Families	12.34	**50.60**	19.85	17.20
	Couples	16.27	11.63	**41.38**	30.71
	Friends	35.82	7.83	16.91	**42.45**

**Table 19:** Earth mover’s distance *C*_*ij*_, non-hierarchical, families with children (in %).

		Colleagues	Families	Couples	Friends
GT	Colleagues	**73.49**	0.76	8.12	17.63
	Families	8.75	**62.30**	19.75	9.20
	Couples	17.77	3.11	**56.69**	22.43
	Friends	36.74	0.60	24.92	**37.73**

### Hierarchical stage-1

#### Results of Bayesian approach for stage-1 of hierarchical recognition

We may see in Table 4, that the correct relation is always identified with higher rate. In addition, in Table 5, friends seem to be the most challenging social relation to identify due to its similarity with colleagues. As reported in Table 2, this is a relatively hard task also for human coders.

#### Results of EMD for stage-1 of hierarchical recognition

Comparing Tables 4 to 6 and Tables 5 to 7, we observe that the results are slightly worse using EMD. Namely, the recognition rates are more fair using the Bayesian approach; and families, couples and friends are recognized with better accuracy.

### Hierarchical stage-2

#### Results of Bayesian approach for stage-2 of hierarchical recognition

From Table 8, it is seen that the correct relation is always identified with higher rate (i.e. the diagonal entries are always higher than the other entries on the same rows.). In addition, leaving colleagues out in stage-1 of hierarchical classification, friends have the best recognition rate due to its similarity to colleagues and difference to families and couples.

#### Results of EMD for stage-2 of hierarchical recognition

Using EMD instead of the Bayesian approach at stage-2 of hierarchical classification, we see that the method fails in attaining always the maximum recognition rate on the diagonal. As shown by Table 2, the “leisure” categories are, even between them, harder to distinguish also for human coders. Furthermore, the confusion between couples and families, which is particularly high in Table 9, is one of the most common also between human coders. This confusion is due to a few reasons. First of all, here the category of families covers all the dyads that are labeled as family, irrespective of their age profile. Therefore, couples that seem to be married are labeled as families, whereas couples that seem to be unmarried are labeled as couples. In order to better indicate the effect of this factor, we applied the same recognition problem to a subset of dyads, where all dyads labeled as families involve at least one member below 15 years old (see Different definition for the family relation section and S6 Appendix).

### Non-Hierarchical

#### Results of Bayesian approach for non-hierarchical recognition

In Table 10, we may see that the correct relation is always identified with a higher rate (i.e. the maximum value on a row is always at the diagonal entry.). Relatively high failure rates are present, when friends are mislabeled as colleagues, and when couples are mislabeled as families. As already stated, the confusion between these categories is present, although at a much lower level, even between human coders (see Table 2).

#### Results of EMD for non-hierarchical recognition

The EMD method fails in attaining maximum recognition rates on the diagonal in the non-hierarchical process (see Table 11). In particular, when compared to the Bayesian method, the confusion between families and couples is particularly high (the method appears to have a strong bias towards couples), while the confusion between colleagues and friends is very similar in the two methods (standard errors are typically of order 1%). Nevertheless, a fair comparison on the trajectory level between the two methods is discussed in Comparing Bayesian approach and functional metric methods section.

In general, a similar discussion to the case in Results of Bayesian approach for stage-2 of hierarchical recognition and Results of EMD for stage-2 of hierarchical recognition sections regarding families and friends, can be made also for Table 11. Namely, families are most often confused with couples due to the assumption of the coders that older couples are more likely to be married and thus be families; and younger couples are more likely to be unmarried and more often labeled as couples. We provide a discussion on this statement in Different definition for the family relation section.

In addition, similar to the reasons of having a significant increase in detection of friends in Tables 8 and 9 in comparison to Tables 5 and 7, the likeness of colleagues and friends undermines the detection of friends.

### Comparing Bayesian approach and functional metric methods

As mentioned in Assessment of the Bayesian method section, contrasting event-based $C_{ij}^{be}$ of Bayesian method to the trajectory-based *C*_*ij*_ of EMD is not a fair comparison. Therefore, we provided a trajectory based evaluation $C_{ij}^{v}$ for the Bayesian method and this section discusses the concerning results.

Table 12 presents the trajectory based assessment of the Bayesian method. It is clear that compared to the event-based confusion assessment, friends suffer a decline in recognition performance and its confusion with colleagues is to a larger extent. A comparison with Table 11 shows that while the Bayesian method is better at distinguishing between families and couples, its performance on distinguishing friends from colleagues is lower than EMD. Furthermore, when evaluated on trajectories, also the Bayesian method fails in attaining maxima on the diagonal for each row (i.e. category).

We suggest that the reason for not sustaining event-level performance in trajectory-level is related to the length of the trajectories. Specifically, in event-based assessment, each event contributes to the results equally regardless of the length of trajectory that it belongs. However, by using the binary-by-trajectory-voting assessment, short and long trajectories contribute to the performance measure defined in Eq 13 with equal weight, making the events from shorter trajectories somewhat more important. Obviously, longer trajectories involve more information on the type of social relation and thus trivializing their significance against short trajectories may cause this degradation in performance.

We can confirm this hypothesis by applying a threshold on trajectory length. For this purpose, we check the median length of trajectories and consider only those trajectories longer than the median length (see Tables 13 and 14). In this manner, we see that all recognition rates of the Bayesian method achieve their maxima on the diagonal, whereas the functional metric method still suffers from the similarity between colleagues and friends; and between families and couples.

### Results of Bayesian approach for *α* ≠ 1

In Tables 15 and 16, we report the results obtained in the $C_{ij}^{be}$, non-hierarchical case by using, respectively, *α* = 0.5 and *α* = 0. By comparing to Table 10, we see that by using *α* ≠ 1 we have a degradation of recognition rates for all categories except couples. In particular, if *α* ≠ 1 is used, the method fails in assigning the correct relation with the highest rate to families and friends.

The improvement in the rate of recognition of couples for *α* = 0 is probably due to the fact that a fixed prior with value 1/4 is assigned to this relation, a value that is much higher than its actual rate of representation (100 couples over 1019 dyads). It is then interesting to see what happens if we directly use the actual rates of representations of each relation as priors. The results, shown in Table 17, suggest that using the rates of representation as priors causes a strong bias towards the most represented classes, and in particular only in the case of colleagues the correct relation is assigned with the highest rate, while friends are often confused with colleagues, and families and couples are mainly confused with friends.

Although we did not explicitly try to search for the value of *α* that provides the best recognition rates, the results of this section suggest that by using *α* ≈ 1, i.e. by updating priors on the basis of the previous estimates on the dyad relation, leads to a better performance. Further details of recognition rates for *α* ≠ 1 are reported in S5 Appendix.

### Different definition for the family relation

As mentioned in Families with children section, we restricted the social relation of families to dyads with at least one member younger than 15 years old and repeated the analysis presented in Hierarchical stage-1 to Non-Hierarchical sections. For the sake of brevity, here we present the results concerning the non-hierarchical method (see Tables 18 and 19), which show that through a better labeling of social relations, it is possible to attain always the highest recognition rate for the correct category.

It may nevertheless be noticed that although the EMD method attains very good recognition for colleagues, families and couples, in the case of friends its correct recognition rate (37.73) exceeds the value of confusion between friends and colleagues (36.74) by a tiny margin. On the other hand, the Bayesian method does not suffer from this problem.

More details may be found in S6 Appendix.

## Conclusions

This work focuses on pedestrian dyads in their ecological environment and proposes a method to identify the type of social relation between their peers. In that respect, we examine the literature on categorization of social relation and find that the approach proposed by Bugental applies to the scenario in focus considerably well. Therefore, we account the four social relation categories of colleagues, families, couples and friends, to be the potential relations between the dyads in our data set, which is recorded in a public space involving a large variety of visitors from different age ranges, with different purpose of visit and backgrounds.

The set is annotated by examining the video footage and the trajectory data obtained from 3D range sensors. Using this ground truth and locomotion information, we first define various observables and establish their discriminating power. Subsequently, we propose two methods, one using Bayesian inference, and another one using a functional metric on probability density functions, to resolve for the social relation.

Our results show that we can always easily distinguish work-oriented dyads (colleagues) from leisure-oriented ones (families, couples, friends). It is also possible, at least using the Bayesian method, to distinguish leisure-oriented dyads between them, when no work-oriented dyad is presented to the algorithm. Nevertheless, the complete recognition of the colleagues, family, couple and friends categories appears to be harder, and although attained by the Bayesian method at the “event” level, it is not attained at the trajectory level (neither by the Bayesian nor by the functional metric method). This is shown to be due to the effect of the difficulty of recognizing short trajectories (with few events). This problem appears to have a considerable effect in particular on the functional metric method, since this approach needs to explicitly build the empirical pdf for the observed trajectory. On the other hand, the independence of the Bayesian approach from the trajectory pdf appears to make it superior in the resolution of the proposed problem. Nevertheless, when we further specified the definition of families by using only trajectories of dyads involving a child, we were able to obtain satisfactory recognition rates also with the functional metric method. Moreover, the Bayesian method attains the best performance when the priors are modified using past outcomes. It may be expected that optimizing on the learning parameter *α* could further increase its performance.

A possible limitation in a real world application of our approach may reside in the fact that pedestrian groups may exhibit non-walking behaviors (i.e. they may stop), and in such situations the present version of our algorithm would not provide an update of recognition rates. Another limitation resides in the environment dependence of the group observable distributions. For example, [7] and [17] show that the observable distribution functions depend on environmental features such as corridor width and crowd densities, and the dependence on other factors such as culture may be speculated. In [1] we analyze the joint effect of relation and density, and the results suggest that, for example, the velocity distribution of colleagues is different enough from those of the other relations to allow recognition up to moderate densities. It is nevertheless probable that to perform recognition in real world settings some environment-specific calibration of the method would be necessary. Despite these limitations, we believe that our approach may contribute both from a theoretical viewpoint and as a first step from a practical viewpoint in the novel field of automatic group relation recognition.

Possible improvements in our method could be related to working with a different or extended observable space, and coping with difficulties in distinguishing similar relations such as couples and families by implementing different learning methods and possibly combining their results to the one of the proposed method. One possibility could also be to investigate methods that could recognize the behavior of groups under specific and relatively rare conditions (“diagnostic events”). Finally, any recognition method would definitely profit from larger and better labeled data sets.

## Supporting information

S1 File Data set. The trajectories of individual pedestrians are provided as a ZIP archive.
(ZIP)

S1 Appendix Observable distributions of work-oriented vs leisure-oriented groups. Empirical distributions similar to those in Empirical observations distributions section are derived for different purpose of visit.
(PDF)

S2 Appendix Justification of conditional independence of observables. Conditional independence of observables is verified using normalized entropy distance.
(PDF)

S3 Appendix Extending Earth Mover’s Distance to multivariate space with independent components. Computation of Earth Mover’s Distance in multivariate space with independent components is elaborated on.
(PDF)

S4 Appendix Alternative measures of difference between pdfs. Two alternative methods, i.e. Kullback-Leibler divergence and Jensen-Shannon divergence, are explained and it is shown that the divergence terms computed by these methods regarding two probability density functions of a multivariate random variable with independent components can be written as the sum of divergences along each dimension.
(PDF)

S5 Appendix Results for the Bayesian method using *α* ≠ 1. Similar analysis of the results to the one in Results section is provided for values of *α* ∈ {0, 0.5}.
(PDF)

S6 Appendix A different definition for the family relation. Restricting the social relation of families to dyads with at least one member younger than 15 years old, we repeat the analysis presented in Hierarchical stage-1 to Non-Hierarchical sections.
(PDF)

S7 Appendix Recognition with varying sizes of training set. We report, for the non-hierarchical, *α* = 1 case, recognition rates obtained by using training sets corresponding to 15% and 50% of the data set.
(PDF)
