# Supplementary material for: Identification of social relation within pedestrian dyads
Source: PLoS One. 2019 Oct 17;14(10):e0223656. doi: 10.1371/journal.pone.0223656 (PMC6797107; doi:10.1371/journal.pone.0223656)
Supplement: S1 Appendix — Empirical distributions similar to those in Empirical observations distributions section are derived for different purpose of visit. (PDF) [file pone.0223656.s002.pdf]

Observable distributions of work-oriented vs leisure-oriented groups

By employing the annotation of the "purpose of visit", dyads which are "work-oriented" and "leisure-oriented" are identified. Since work-oriented dyads coincide almost perfectly with colleagues, the collection of families, couples, friends are considered to constitute a complementary relation to "work-oriented", denoted by "leisure-oriented". In this manner, the four observables compare for "work-oriented" and "leisure-oriented" as given in Figures 1-4. Clearly, δ and v_g turn out to be the most distinguishing observables, even thought ω and η are also shown to have a statistically significant effect.

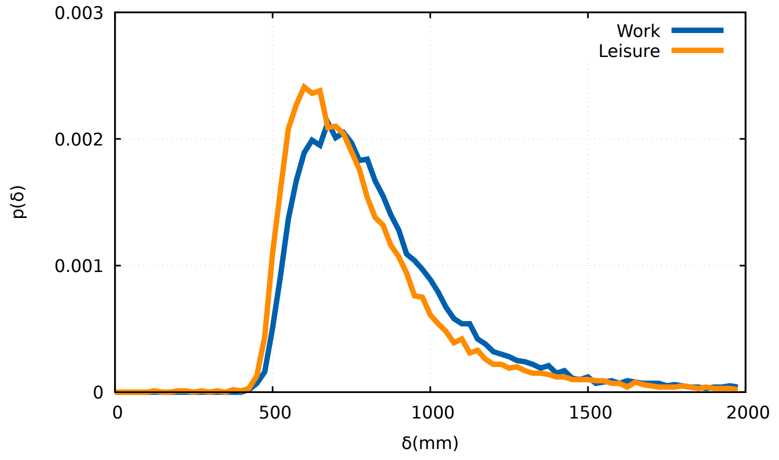

Figure 1: Pdf of δ used in hierarchical stage-1.

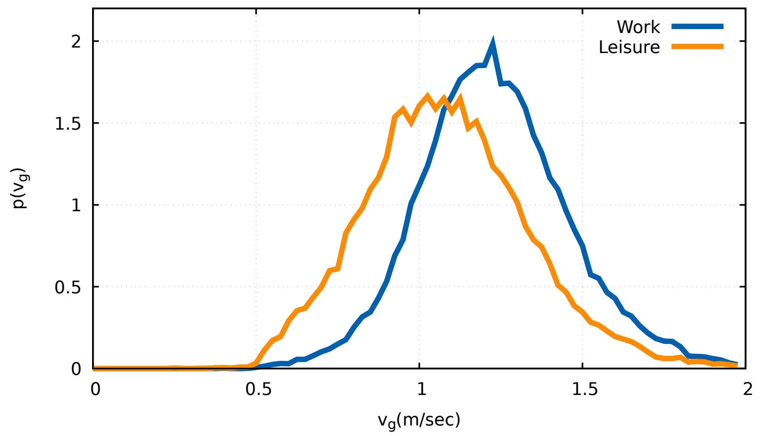

Figure 2: Pdf of v_g used in hierarchical stage-1.

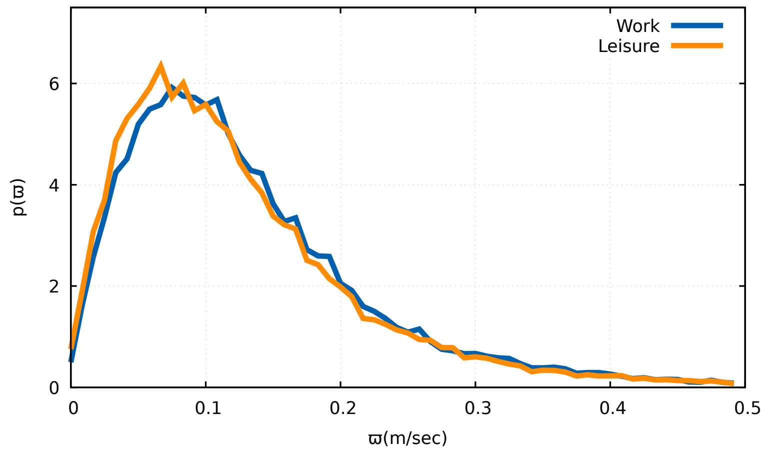

Figure 3: Pdf of ω used in hierarchical stage-1.

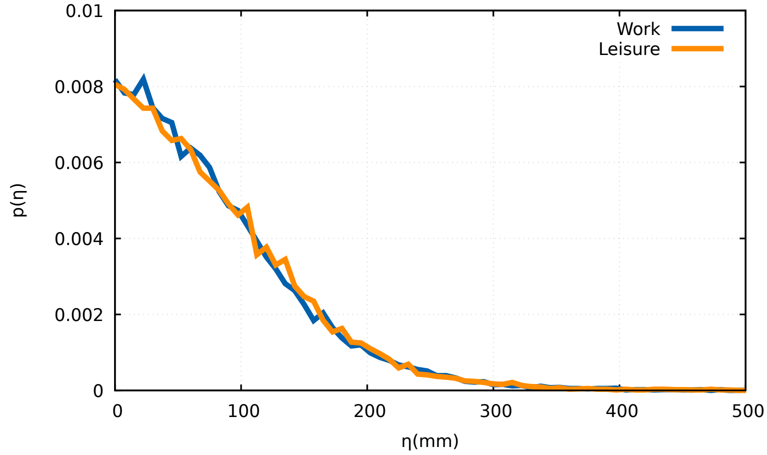

Figure 4: Pdf of η used in hierarchical stage-1.
