# Supplementary material for: Identification of social relation within pedestrian dyads
Source: PLoS One. 2019 Oct 17;14(10):e0223656. doi: 10.1371/journal.pone.0223656 (PMC6797107; doi:10.1371/journal.pone.0223656)
Supplement: S2 Appendix — Conditional independence of observables is verified using normalized entropy distance. (PDF) [file pone.0223656.s003.pdf]

Justification of conditional independence of observables

Operating in the full 4-D space of observables (δ, v_g, ω, and η), yields an overwhelmingly large number of bins. Since our data points are clearly not sufficient to calibrate 4-D empirical observables, we opt for using 1-D observable spaces. However, this choice begs a justification for conditional independence of observables. To verify this hypothesis, we adopt an information theoretic approach.

Principally, the entropy distance of two random variables as Θ and Δ is defined as ,

$$D_H(\Theta, \Delta) = H(\Theta, \Delta) - I(\Theta; \Delta) \tag{1}$$

where $H(\Theta, \Delta)$ and $I(\Theta, \Delta)$ are respectively the joint entropy and mutual information of these variables [1].

In explicit terms, joint entropy is

$$H(\Theta, \Delta) = -\sum_{i,j} p(\theta_i, \delta_j) \log_2(p(\theta_i, \delta_j)), \tag{2}$$

whereas the mutual information is defined as

$$I(\Theta; \Delta) = \sum_{i,j} p(\theta_i, \delta_j) \log_2 \left(\frac{p(\theta_i, \delta_j)}{p(\theta_i) p(\delta_j)} \right). \tag{3}$$

On the other hand, the normalized entropy distance defined as

$$D(\Theta, \Delta) = \frac{D_H(\Theta, \Delta)}{H(\Theta, \Delta)}, \tag{4}$$

is more useful for our purposes since it is a *true* metric. Namely, as outlined in [2] and more elborately treated in [3], it is non-negative, symmetric and it satisfies the triangle inequality. Specifically, for uncorrelated variables, the normalized entropy distance $D(\Theta, \Delta)$ should be 1, and closer to 0 for correlated ones.

Table 1 shows normalized entropy distance values between pairs of observables for two sample subset of dyads belonging to different social relations. The subsets are randomly chosen and consist 30% of the entire observations (i.e. dyads) relating any social relation. This random selection process is repeated 50 times to test the resilience of independence to varying observation sets. The mean values of normalized entropy distance concerning these 50 runs are illustrated in Table 1, whereas the standard deviations all turn out to be smaller than 10^{-2} and are thus omitted[1]. The results being always higher than 0.94, the conditional independence hypothesis can be considered as reasonable.

[1]Since the matrices in Table 1 are symmetric, only the upper triangular part is presented.

Table 1: Normalized entropy distance for all possible pairs of observables in all social relation categories.

Colleagues

	δ	v_g	ω	η
δ	0	0.98	0.98	0.98
v_g	-	0	0.98	0.98
ω	-	-	0	0.98
η	-	-	-	0

Families

	δ	v_g	ω	η
δ v_g	0	0.98	0.97	0.97
v_g	-	0	0.98	0.97
ω	-	-	0	0.95
η	-	-	-	0

Couples

	δ	v_g	ω	η
δ	0	0.97	0.95	0.96
v_g	-	0	0.96	0.96
ω	-	-	0	0.94
η	-	-	-	0

Friends

	δ	v_g	ω	η
δ	0	0.99	0.98	0.98
v_g	-	0	0.98	0.98
ω	-	-	0	0.98
η	-	-	-	0

References

[1] MacKay DJ. Information theory, inference and learning algorithms. Cambridge University Press; 2017. Available from: `https://www.inference.org.uk/itprnn/book.pdf`.

[2] Aghagolzadeh M, Soltanian-Zadeh H, Araabi B, Aghagolzadeh A. A hierarchical clustering based on mutual information maximization. In: Proceedings of IEEE international conference on image processing. vol. 1. IEEE; 2007. p. I – 277–I – 280.

[3] Li M, Chen X, Li X, Ma B, Vitányi P. The similarity metric. arXiv preprint arXiv:cs/0111054. 2004;.
