# Supplementary material for: Identification of social relation within pedestrian dyads
Source: PLoS One. 2019 Oct 17;14(10):e0223656. doi: 10.1371/journal.pone.0223656 (PMC6797107; doi:10.1371/journal.pone.0223656)
Supplement: S3 Appendix — Computation of Earth Mover’s Distance in multivariate space with independent components is elaborated on. (PDF) [file pone.0223656.s004.pdf]

Extending Earth Mover's Distance to multivariate space with independent components

The EMD process can be visualized as filling holes by moving piles of dirt. Assume that P and Q denote two pdfs, and that a proper metric, named *ground distance* is defined to measure the distance between the bins i and j (It is common practice to use the Euclidean distance from i to j as the ground distance. Namely, in the 1D case this reduces to $|i - j|$.) Suppose also that a flow $f(i, j)$ is applied to morph P to Q, namely a (signed) quantity is subtracted from $P(i)$ and added to $Q(j)$ in the process of making the P and Q distributions more similar.

In this contest, EMD can be formulated and solved essentially as a transportation problem [1]. Namely, EMD aims at finding the amount of flow f that minimizes the overall cost of morphing P to Q. Explicitly, the work required to morph P to Q (or viceversa) *given an explicit flow* f is,

$$\sum_i \sum_j f(i,j)d(i,j), \tag{1}$$

where $f(i, j)$ and $d(i, j)$ are respectively, the flow and the ground distance between $P(i)$ and $Q(j)$ [2]. By solving for the optimal flow and normalizing it with the total flow, EMD is described as,

$$EMD(P,Q) = \frac{\min_f \sum_i \sum_j f(i,j)d(i,j)}{\sum_i \sum_j f(i,j)}.$$

The normalization operation in the above equation yields the average distance traveled by unit weight under the optimal flow.

When computing the EMD between two discrete histograms defined on the same array of bins $i = 0, ..., N - 1$, the following algorithm may be used

$$\begin{aligned} \text{EMD}_0 &= 0, \\ \text{EMD}_{i+1} &= Q_i - P_i + \text{EMD}_i, \\ \text{EMD} &= \sum_{i=1}^{N} |\text{EMD}_i|. \end{aligned} \tag{2}$$

To operate with the EMD in a multidimensional setting under the assumption of independence, we proceed as follows. Without loss of generality, we initially restrict ourselves to the 2-D case.

For each metric defined on a probability distribution space with 2 random variables as α_1 and α_2, we have,

$$d(f_{\boldsymbol{\alpha}}, g_{\boldsymbol{\alpha}}) = d(f_{\alpha_1} f_{\alpha_2}, g_{\alpha_1} g_{\alpha_2}) \le d(f_{\alpha_1} f_{\alpha_2}, f_{\alpha_1} g_{\alpha_2}) + d(f_{\alpha_1} g_{\alpha_2}, g_{\alpha_1} g_{\alpha_2}) \tag{3}$$

assuming independence of α_1 and α_2.

If the distance on probability distributions is defined in such a way that a common multiplicative term in one of the variables factorizes and can be integrated to 1 and disappear, namely

$$d(X_1(x)Y(y), X_2(x)Y(y)) = d(X_1(x), X_2(x)), \tag{4}$$

then we have

$$d(f_{\boldsymbol{\alpha}}, g_{\boldsymbol{\alpha}}) \leq d(f_{\alpha_2}, g_{\alpha_2}) + d(f_{\alpha_1}, g_{\alpha_1}),$$

and the distance between the two 2-D distributions is bounded by the sum of the distances along the two components. In the case of EMD, Equation 4 corresponds to saying that

$$\begin{aligned} \min(\text{Cost from } F_i^1 F_j^2 \text{ to } G_i^1 F_j^2) &= \left(\sum_j F_j^2\right) \min(\text{Cost from } F_i^1 \text{ to } G_i^1) \\ &= \min(\text{Cost from } F_i^1 \text{ to } G_i^1) \end{aligned} \tag{5}$$

The generalization to the n-D case is trivially obtained by using,

$$\begin{aligned} d(f_{\boldsymbol{\alpha}}, g_{\boldsymbol{\alpha}}) = d(f_{\alpha_1} f_{\alpha_2} f_{\alpha_3}, g_{\alpha_1} g_{\alpha_2} g_{\alpha_3}) &\leq d(f_{\alpha_1} f_{\alpha_2} f_{\alpha_3}, f_{\alpha_1} f_{\alpha_2} g_{\alpha_3}) \\ &+ d(f_{\alpha_1} f_{\alpha_2} g_{\alpha_3}, f_{\alpha_1} g_{\alpha_2} g_{\alpha_3}) + d(f_{\alpha_1} g_{\alpha_2} g_{\alpha_3}, g_{\alpha_1} g_{\alpha_2} g_{\alpha_3}), \end{aligned}$$

and so on.

Let us now see why Equation 5 holds. Using the algorithm to compute the discrete 1-D EMD as written in eq. 2, we have

$$\begin{aligned} \text{EMD}_0 &= 0, \\ \text{EMD}_{i+1} &= F_i^1 - G_i^1 + \text{EMD}_i, \\ \text{EMD} &= \sum_i |\text{EMD}_i|. \end{aligned} \tag{6}$$

Namely, if we have a difference $F_0^1 - G_0^1 = \Delta_0$, this quantity is displaced to the first bin k such that $F_k^1 - G_k^1 = \Delta_k$ with $\Delta_0 \Delta_k < 0$. If $|\Delta_0| \leq |\Delta_k|$, then Δ_0 is used to fill the difference in k and contributes with $|\Delta_0| k$ to the EMD. Otherwise, the remaining quantity $|\Delta_0 + \Delta_k|$ is displaced to the following bin l such that $\Delta_0 \Delta_l < 0$, and so on. The logic is that we are moving Δ_0 to the closest bin with a different Δ sign.

Now, in the 2-D case of Equation 5, we have $\Delta_{0,j} = (F_0^1 - G_0^1) F_j$. Since all the terms on a given row have $|\Delta_{i,j} \Delta_{i,k}| = (F_i^1 - G_i^1)^2 F_j F_k > 0$, the closest term to $\Delta_{0,j}$ with $\Delta_{0,j} \Delta_{i,l} < 0$ will be found moving on a straight line on a given column, namely it will be $\Delta_{k,j}$, and so on. We may see now that displacements along rows are never

performed, and the 2-D algorithm becomes,

$$\begin{aligned}\text{EMD}_0 &= 0,\\ \text{EMD}_{i+1} &= (F_i^1 - G_i^1)\left(\sum_j F_j^2\right) + \text{EMD}_i = F_i^1 - G_i^1 + \text{EMD}_i,\\ \text{EMD} &= \sum_i |\text{EMD}_i|.\end{aligned} \tag{7}$$

This property of independent variables also allows us to avoid a subtle problem in handling EMD in our multi-dimensional space. The 1-D EMD is based on a metric (ground distance) that evaluates the contribution of the quantity Δ moved between bins i and j to EMD as $|\Delta||j-i|$. Namely, the displacement is measured linearly as $|j-i|$. How should we measure the displacement between bins (i,k) and (j,l) corresponding to variables of a different nature, such as velocity and distance? We have just shown that this problem is not relevant in the computation of the upper bound Equation 3, since it is not relevant in the computation of Equation 5. Indeed, for any ground distance such that the distance between bins in a column is still linear,

$$\text{ground}((i,k),(j,k)) \propto |j-i|,$$

we will have

$$\text{ground}((i,k),(j,k)) \leq \text{ground}((i,k),(j,l)),$$

i.e. minimum displacements are obtained moving along columns (or rows). Indeed, if we had,

$$\text{ground}((i,k),(j,l)) < \text{ground}((i,k),(j,k)), \tag{8}$$

we would obtain by symmetry (implied by the linearity of displacements on row and columns)

$$\text{ground}((i,k),(j,l)) + \text{ground}((j,l),(2j-i,k)) < \text{ground}((i,k),(2j-i,k)), \tag{9}$$

in contradiction with the triangular inequality.

References

[1] Lieberman GJ, Hillier F. Introduction to Mathematical Programming. McGraw-Hill; 1995.

[2] Wei J. On Markov Earth Mover's Distance. International journal of image and graphics. 2014;14(04):1450016.
