# Supplementary material for: Identification of social relation within pedestrian dyads
Source: PLoS One. 2019 Oct 17;14(10):e0223656. doi: 10.1371/journal.pone.0223656 (PMC6797107; doi:10.1371/journal.pone.0223656)
Supplement: S4 Appendix — Two alternative methods, i.e. Kullback-Leibler divergence and Jensen-Shannon divergence, are explained and it is shown that the divergence terms computed by these methods regarding two probability density functions of a multivariate random variable with independent components can be written as the sum of divergences along each dimension. (PDF) [file pone.0223656.s005.pdf]

Alternative measures

As shown in detail in S3 Appendix[1], for the 4-D Earth Mover's distance, we are able, under the assumption of statistical independence of the variables, to provide only an upper bound and not a complete computation. For theoretical completeness, we introduce here two other ways to compute the difference between probability density functions for which such a complete computation is possible, namely the Kullback-Leiber and Jensen-Shannon divergences. Despite this theoretical property, these two divergences do not provide results as good as those obtained using the Earth Mover's Distance.

Kullback-Leibler divergence

Assuming that P and Q are two probability distributions, the KullbackLeibler divergence from Q to P is denoted by $D_{KL}(P\|Q)$ and evaluates the difference between distributions P and Q [1].

Although KullbackLeibler divergence is often confused as a way of measuring the *distance* between two probability distributions, it is, mathematically speaking, not a (distance) metric, since it is not symmetric,

$$D_{KL}(P\|Q) \neq D_{KL}(Q\|P),$$

neither does it satisfy the triangular inequality, i.e. it is not in general true that

$$D_{KL}(P\|Q) \leq D_{KL}(P\|R) + D_{KL}(R\|Q),$$

where R denotes a (third) probability distribution.

However, it is not uncommon to use Kullback-Leibler divergence in machine learning as a way to evaluate the information gain achieved, if Q is used instead of P. Explicitly, it is defined as

$$D_{KL}(P\|Q) = \Sigma_i \left(P(i) \log \left(\frac{P(i)}{Q(i)} \right) \right). \tag{1}$$

An inherent issue with implementations of Kullback-Leibler divergence is related to the empty bins in the probability mass functions. Namely, provided that some values are never observed in the data set, the relating probability values turn out to be 0 due to the empirical approach. While $P(i) = 0$ does not represent a problem, since $\lim_{x\to 0^+} x \log x = 0$ and thus the corresponding terms may be set to 0; terms with $Q(i) = 0$ are undefined.

Nevertheless, in practice there are several workarounds for such cases. Here, we choose to apply a small offset of 10^{-10} on all values in the empirical pdf. Since the number of observations in our data set is $\approx 10^4$, for any $Q(i) \neq 0$ we have $Q(i) \gg 10^{-10}$, and thus adding the offset removes the diverging terms without modifying the distribution $Q(i)$ in a significant way[2].

[1] Refer to main track for the link.

[2] Obviously, after adding the offset, the distribution $Q(i)$ is newly normalized in order to preserve $\sum_i Q(i) = 1$.

In addition, although Kullback-Leibler divergence is often utilized on univariate random variables, adaptation of Equation 1 to the multivariate case is straightforward, particularly thanks to the independence relation. Namely, as explained in the forthcoming sections, Kullback-Leibler divergence of two probability density functions concerning a multivariate random variable with independent components can be written as the sum of divergences along each dimension.

Jensen-Shannon divergence

As explained in the previous section, the term in Equation 1 has an asymmetric nature, which disqualifies it from being a *true* metric. In order to evaluate the impact of this asymmetry on performance, we propose contrasting the recognition results obtained using Kullback-Leibler divergence to those obtained employing Jensen-Shannon divergence.

In particular, Jensen-Shannon divergence is based on Kullback-Leibler divergence, but it is symmetric and has always a finite value. Namely, a reference distribution M is derived from P and Q appearing in Equation 1, such that,

$$M(i) = \frac{1}{2}\left(P(i) + Q(i)\right). \tag{2}$$

By expressing the divergence of P and Q as the sum of their individual (Kullback-Leibler) divergences in relation to the (same) reference distribution in Equation 2, the Jensen-Shannon divergence term $D_{JS}\left(P\|Q\right)$ boils down to,

$$D_{JS}\left(P\|Q\right) = \frac{1}{2}D_{KL}\left(P\|M\right) + \frac{1}{2}D_{KL}\left(Q\|M\right). \tag{3}$$

It may be shown that, provided base 2 is used for the logarithm, D_{JS} assumes values[3] in $[0, 1]$. Furthermore, $\sqrt{D_{JS}}$ is a proper metric (i.e. it satisfies also the triangular inequality).

Concerning the extension of the univariate case onto multivariate random variables, the proof provided in the next section can be generalized to the Jensen-Shannon divergence. Namely, Jensen-Shannon divergence of two probability density functions concerning a multivariate random variable with independent components can be written as the sum of divergences along each dimension. The details are found in the last section of this document.

Extending Kullback-Leibler divergence to multivariate space with independent components

Remember that Kullback-Leibler divergence of two discrete probability distributions as P and Q is defined in Equation 1 quantifies the divergence "from P to Q". Without loss

[3]The only case, which could lead to a problem in Equation 3, is when a given bin is empty in both distributions (i.e. in both P and Q). However, from a practical point of view, this does not pose a problem for computing the divergence, since it is immanent to disregard the "unobserved" values.

of generality, let us assume that $\boldsymbol{\alpha}$ is a multivariate random variable, with components α_1 and α_2,

$$\boldsymbol{\alpha} = \begin{bmatrix}\alpha_1 & \alpha_2\end{bmatrix}^T.$$

Let us denote the multivariate probability distribution of $\boldsymbol{\alpha}$ by $f_{\boldsymbol{\alpha}}$. Provided that α_1 and α_2 are independent, $f_{\boldsymbol{\alpha}}$ can be decomposed into the following product,

$$f_{\boldsymbol{\alpha}} = f_{\alpha_1} f_{\alpha_2}.$$

Suppose $g_{\boldsymbol{\alpha}}$ is another joint probability distribution relating the same random variable $\boldsymbol{\alpha}$ and is also decomposed as $g_{\boldsymbol{\alpha}} = g_{\alpha_1} g_{\alpha_2}$.

According to Equation 1 and under the assumption of independence of α_1 and α_2, Kullback-Leibler divergence from $g_{\boldsymbol{\alpha}}$ to $f_{\boldsymbol{\alpha}}$ can be written as follows,

$$D_{KL}\left(f_{\boldsymbol{\alpha}} \| g_{\boldsymbol{\alpha}}\right) = \sum_{\alpha_{1,2}} \left(f_{\alpha_1} f_{\alpha_2} \log\left(\frac{f_{\alpha_1} f_{\alpha_2}}{g_{\alpha_1} g_{\alpha_2}}\right)\right).$$

Here the logarithmic term can be broken down as,

$$D_{KL}\left(f_{\boldsymbol{\alpha}} \| g_{\boldsymbol{\alpha}}\right) = \sum_{\alpha_{1,2}} \left(f_{\alpha_1} f_{\alpha_2} \left[\log\left(\frac{f_{\alpha_1}}{g_{\alpha_1}}\right) + \log\left(\frac{f_{\alpha_2}}{g_{\alpha_2}}\right)\right]\right).$$

The summation terms can be rearranged such that

$$D_{KL}\left(f_{\boldsymbol{\alpha}} \| g_{\boldsymbol{\alpha}}\right) = \sum_{\alpha_1} f_{\alpha_1} \sum_{\alpha_2} \left(f_{\alpha_2} \log\left(\frac{f_{\alpha_2}}{g_{\alpha_2}}\right)\right) + \sum_{\alpha_2} f_{\alpha_2} \sum_{\alpha_1} \left(f_{\alpha_1} \log\left(\frac{f_{\alpha_1}}{g_{\alpha_1}}\right)\right).$$

Here the inner summation terms can be identified as Kullback-Leibler divergences on univariate random variables α_2 and α_1, respectively,

$$D_{KL}\left(f_{\boldsymbol{\alpha}} \| g_{\boldsymbol{\alpha}}\right) = \sum_{\alpha_1} f_{\alpha_1} D_{KL}\left(f_{\alpha_2} \| g_{\alpha_2}\right) + \sum_{\alpha_2} f_{\alpha_2} D_{KL}\left(f_{\alpha_1} \| g_{\alpha_1}\right).$$

By moving these divergence terms out of summation, we obtain,

$$D_{KL}\left(f_{\boldsymbol{\alpha}} \| g_{\boldsymbol{\alpha}}\right) = D_{KL}\left(f_{\alpha_2} \| g_{\alpha_2}\right) \sum_{\alpha_1} f_{\alpha_1} + D_{KL}\left(f_{\alpha_1} \| g_{\alpha_1}\right) \sum_{\alpha_2} f_{\alpha_2}.$$

Note that the terms $\sum_{\alpha_1} f_{\alpha_1}$ and $\sum_{\alpha_2} f_{\alpha_2}$ are the integrals of the pdfs and thus integrate to 1. Therefore, they can simply be omitted,

$$D_{KL}\left(f_{\boldsymbol{\alpha}} \| g_{\boldsymbol{\alpha}}\right) = D_{KL}\left(f_{\alpha_2} \| g_{\alpha_2}\right) + D_{KL}\left(f_{\alpha_1} \| g_{\alpha_1}\right).$$

This result can be generalized to any number of dimensions of $\boldsymbol{\alpha}$

$$\boldsymbol{\alpha} = \begin{bmatrix}\alpha_1 & \alpha_2 & \dots & \alpha_n\end{bmatrix}^T,$$

as follows,

$$D_{KL}\left(f_{\boldsymbol{\alpha}} \| g_{\boldsymbol{\alpha}}\right) = \sum_{i=1}^{n} D_{KL}\left(f_{\alpha_i} \| g_{\alpha_i}\right).$$

Thus, Kullback-Leibler divergence of two probability density functions concerning a multivariate random variable with independent components can be written as the sum of divergences along each dimension.

Extending Jensen-Shannon divergence to multivariate space with independent components

The proof provided in the previous section could be immediately extended to D_{JS} if the reference distribution

$$h_{\boldsymbol{\alpha}} = \frac{1}{2}\left(f_{\boldsymbol{\alpha}} + g_{\boldsymbol{\alpha}}\right), \tag{4}$$

had independent variables. Nevertheless, following the notation of previous section, we see that

$$h_{\boldsymbol{\alpha}} = \frac{1}{2}\left(f_{\alpha_1} f_{\alpha_2} + g_{\alpha_1} g_{\alpha_2}\right), \tag{5}$$

cannot in general be factorized. The solution is to define the D_{JS} for multivariate space with independent components with respect to a factorized reference distribution,

$$h^f_{\boldsymbol{\alpha}} = \frac{1}{4}\left(f_{\alpha_1} + g_{\alpha_1}\right)\left(f_{\alpha_2} + g_{\alpha_2}\right). \tag{6}$$

Using this definition, we still have a divergence with the same properties as D_{JS}, for which the divergence of two probability density functions concerning a multivariate random variable with independent components can be written as the sum of divergences along each dimension.

Results relating alternative measures

We see that both using Kullback-Leibler divergence and Jensen-Shannon divergence acceptable recognition rates are obtained only at stage-1 of hierarchical recognition.

Hierarchical stage-1

Results of Kullback-Leibler divergence for stage-1 of hierarchical recognition

Table 1: Kullback-Leibler divergence, hierarchical stage-1, $\alpha = 1$ (in %).

		Work	Leisure
Ground truth	Work	**81.30**	18.70
	Leisure	41.94	**58.06**

Table 2: Kullback-Leibler divergence, hierarchical stage-1, $\alpha = 1$ (in %) with detailed confusion rates.

		Work	Leisure
Ground truth	Colleagues	**81.30**	18.70
	Families	35.14	**64.86**
	Couples	35.34	**64.66**
	Friends	49.04	**50.96**

Results of Jensen-Shannon divergence for stage-1 of hierarchical recognition

Table 3: Jensen-Shannon divergence, hierarchical stage-1, $\alpha = 1$ (in %).

		Work	Leisure
Ground truth	Work	**78.72**	21.28
	Leisure	39.57	**60.43**

Table 4: Jensen-Shannon divergence, hierarchical stage-1, $\alpha = 1$ (in %) with detailed confusion rates.

		Work	Leisure
Ground truth	Colleagues	**78.72**	21.28
	Families	35.80	**64.20**
	Couples	30.11	**69.89**
	Friends	45.32	**54.68**

Hierarchical stage-2

Results of Kullback-Leibler divergencefor stage-2 of hierarchical recognition

Table 5: Kullback-Leibler divergence, hierarchical stage-2, $\alpha = 1$ (in %).

		Families	Couples	Friends
Ground truth	Families	12.81	**67.76**	19.43
	Couples	5.09	**80.63**	14.29
	Friends	6.75	**73.16**	20.10

Results of Jensen-Shannon divergence for stage-2 of hierarchical recognition

Table 6: Jensen-Shannon divergence, hierarchical stage-2, $\alpha = 1$ (in %).

		Families	Couples	Friends
Ground truth	Families	33.54	**34.93**	31.52
	Couples	18.46	**52.63**	28.91
	Friends	21.55	**40.28**	38.17

Non-Hierarchical

Results of Kullback-Leibler divergence for non-hierarchical recognition

Table 7: Kullback-Leibler divergence, non-hierarchical, $\alpha = 1$ (in %).

		Colleagues	Families	Couples	Friends
Ground truth	Colleagues	**70.45**	1.96	24.33	3.25
	Families	27.28	13.99	**50.81**	7.92
	Couples	24.69	6.66	**61.60**	7.06
	Friends	37.21	7.70	**49.68**	5.41

Results of Jensen-Shannon divergence for non-hierarchical recognition

Table 8: Jensen-Shannon divergence divergence, non-hierarchical, $\alpha = 1$ (in %).

		Colleagues	Families	Couples	Friends
Ground truth	Colleagues	**76.53**	6.26	12.37	4.83
	Families	**32.19**	30.28	30.28	7.26
	Couples	28.14	17.29	**44.74**	9.83
	Friends	**40.66**	20.26	31.00	8.08

References

[1] S. Kullback and R. A. Leibler, "On information and sufficiency," *The Annals of Mathematical Statistics*, vol. 22, no. 1, pp. 79–86, 1951.
