# Supplementary material for: Identification of social relation within pedestrian dyads
Source: PLoS One. 2019 Oct 17;14(10):e0223656. doi: 10.1371/journal.pone.0223656 (PMC6797107; doi:10.1371/journal.pone.0223656)
Supplement: S5 Appendix — Similar analysis of the results to the one in Results section is provided for values of α ∈ {0, 0.5}. (PDF) [file pone.0223656.s006.pdf]

Results for the Bayesian method using $\alpha \neq 1$

Comparing to the results reported in Results of Bayesian approach for stage-1 of hierarchical recognition section of main track to the ones reported in this document, we can say that both in the hierarchical and non-hierarchical methods, using a high α value yields in general better results (at the cost of a slight decrease in identification of couples).

Hierarchical stage-1

Using C_{ij}^{be} as a decision criterion at stage-1 of the hierarchical approach, having a non-zero learning rate (i.e. α) introduces an improvement in identification rates (see Table 3 of main track and Tables 1, 3). Moreover, we can see that similar to Table 5 of the main track, friends are still the category with the lowest recognition rate in Tables 2 and 4.

Table 1: Binary-by-event C_{ij}^{be}, hierarchical stage-1, $\alpha = 0.5$.

		Work	Leisure
Ground truth	Work	**74.02**	25.98
	Leisure	36.04	**63.96**

Table 2: Binary-by-event C_{ij}^{be}, hierarchical stage-1, $\alpha = 0.5$ with detailed confusion rates.

		Work	Leisure
Ground truth	Colleagues	**74.02**	25.98
	Families	27.08	**72.92**
	Couples	24.57	**75.43**
	Friends	46.23	**53.77**

Table 3: Binary-by-event C_{ij}^{be}, hierarchical stage-1, $\alpha = 0$ (in %).

		Work	Leisure
Ground truth	Work	**70.51**	29.49
	Leisure	36.88	**63.12**

Table 4: Binary-by-event C_{ij}^{be}, hierarchical stage-1, $\alpha = 0$ (in %) with detailed confusion rates.

		Work	Leisure
Ground truth	Colleagues	**70.51**	29.49
	Families	28.83	**71.17**
	Couples	27.13	**72.87**
	Friends	45.83	**54.17**

Hierarchical stage-2

Using C_{ij}^{be} in stage-2, friends are detected with an improved rate regardless of the α value, since colleagues are discarded in stage-1. Having a learning rate of $\alpha = 1$ seems to degrade the detection rate of couples, while it helps to better identify families and friends (see Table 7 of main track and and Tables 5, 6).

Table 5: Binary-by-event C_{ij}^{be}, hierarchical stage-2, $\alpha = 0.5$ (in %).

		Families	Couples	Friends
Ground truth	Families	32.07	32.16	**35.76**
	Couples	20.10	**48.39**	31.51
	Friends	16.17	22.34	**61.49**

Table 6: Binary-by-event C_{ij}^{be}, hierarchical stage-2, $\alpha = 0$ (in %).

		Families	Couples	Friends
Ground truth	Families	31.59	30.97	**37.16**
	Couples	21.15	**47.44**	31.42
	Friends	17.71	25.17	**57.41**

Non-Hierarchical

In the non-hierarchical approach with C_{ij}^{be}, the overall identification rates seem to profit from a higher learning rate (see Table 9 of main track and and Tables 7, 8). In particular, $\alpha = 1$ attains, between the tested values, maximum recognition values on the diagonal. This shows the importance of using previous detection results to modify the priors. It

may be expected that the best recognition rates are attained for $0.5 < \alpha < 1$, although this optimization problem is left for future work.

Table 7: Binary-by-event C_{ij}^{be}, non-hierarchical, $\alpha = 0.5$ (in %).

		Colleagues	Families	Couples	Friends
Ground truth	Colleagues	**65.78**	7.55	11.46	15.21
	Families	20.12	29.11	**31.11**	19.65
	Couples	18.71	17.34	**47.75**	16.20
	Friends	**37.67**	13.07	21.35	27.91

Table 8: Binary-by-event C_{ij}^{be}, non-hierarchical, $\alpha = 0$ (in %).

		Colleagues	Families	Couples	Friends
Ground truth	Colleagues	**59.67**	8.42	13.80	18.11
	Families	20.46	27.58	**30.69**	21.26
	Couples	19.27	17.75	**45.25**	17.72
	Friends	**36.22**	13.23	22.05	28.50
