# Supplementary material for: Identification of social relation within pedestrian dyads
Source: PLoS One. 2019 Oct 17;14(10):e0223656. doi: 10.1371/journal.pone.0223656 (PMC6797107; doi:10.1371/journal.pone.0223656)
Supplement: S6 Appendix — Restricting the social relation of families to dyads with at least one member younger than 15 years old, we repeat the analysis presented in Hierarchical stage-1 to Non-Hierarchical sections. (PDF) [file pone.0223656.s007.pdf]

A different definition for the family relation

As mentioned in Families with children section of the main track, we restricted the social relation of families to dyads with at least one member younger than 15 years old and repeated the analysis presented in Hierarchical stage-1 section to Non-Hierarchical section of main track. Even though we evaluated all assessment methods and for each value of α, for the sake of brevity, we present only the assessments, which provide a direct correspondence to the results presented in Hierarchical stage-1 section and Hierarchical stage-2 section of main track. The results using this new definition of families corresponding to those of Non-Hierarchical section of main track have already been presented in Different definition for the family relation section of the main track.

Observable distributions

First of all, we present the distribution of the four observables for the new definition of families (i.e. families with children) as in Figures 1, 2, 3, 4. Note that the curves for colleagues, couples, and friends in these figures are the same as the ones in Figures 3, 4, 5, and 6 of main track, and those of families are the only curves subject to a modification.

Comparing Figure 3 of main track and Figure 1, it can be seen that δ has its peak around a lower value and with a lighter tail, whereas v_g observes a peak (assuming a smoothing) in Figure 2 around more or less the same value as in Figure 4 of main track but with a higher variation. As for ω, the difference between Figure 5 of main track and 3, is quite small but we can say that the peak is somewhat at the same location but the tail gets slightly fatter. The most obvious difference is of course in η, where the height difference between the adult and child can be clearly observed over the entire spectrum of η in Figure 4.

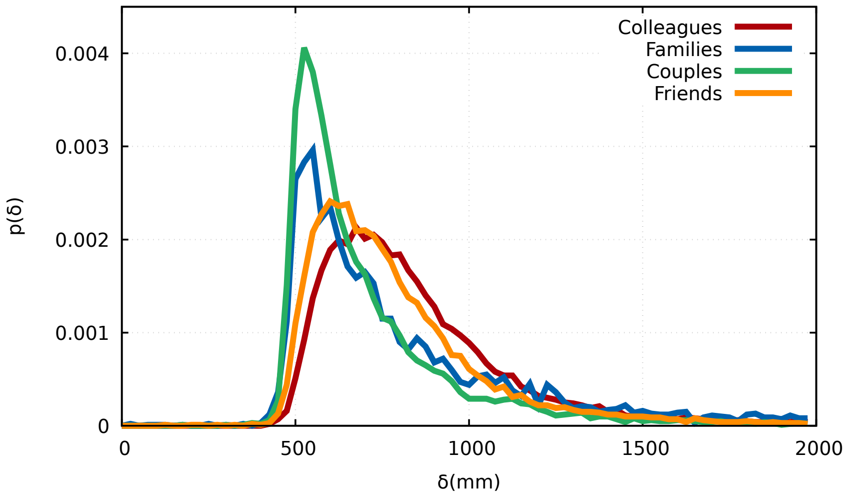

Figure 1: Empirical distribution of interpersonal distance δ, where families are restricted to dyads with at least one member under 15.

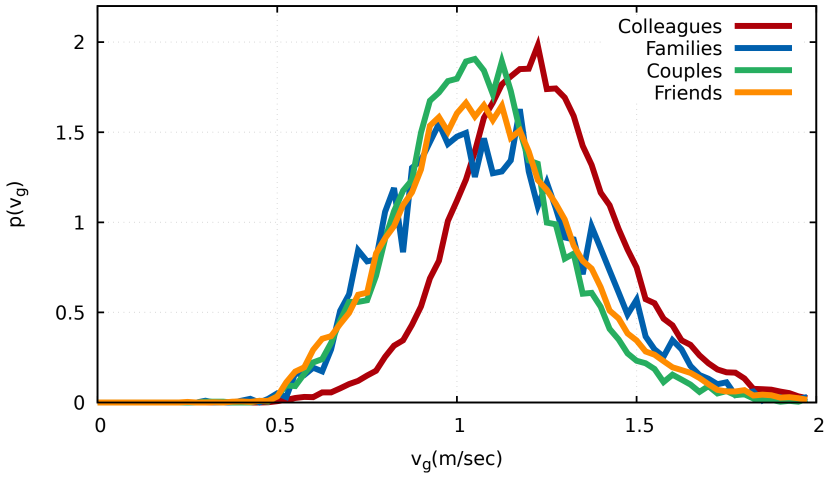

Figure 2: Empirical distribution of group velocity v_g, where families are restricted to dyads with at least one member under 15.

Hierarchical stage-1

We observe that for both methods although there is a minute decline in performance concerning stage-1 of hierarchical recognition, we have a strong improvement both for stage-2 of hierarchical recognition and for non-hierarchical recognition. For the Bayesian method, compare Tables 1 and 5 of this document, Table 14 of main track to Tables 3, 7, 9 of main track; and for EMD compare Tables 3 and 6 of this document and Table 15 of the main track to Tables 5, 8, and 10 of the main track, respectively.

Bayesian approach

Table 1: Binary-by-event C_{ij}^{be}, hierarchical stage-1, families with children, $\alpha = 1$ (in %).

		Work	Leisure
Ground truth	Work	**74.13**	25.87
	Leisure	32.02	**67.98**

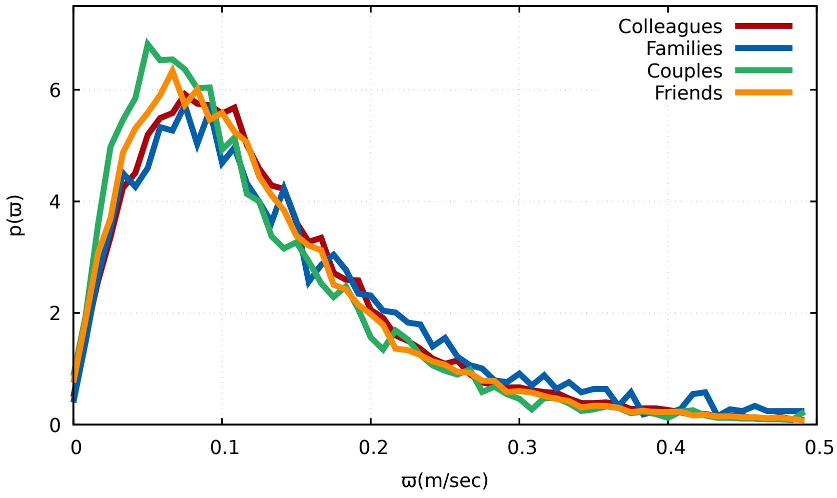

Figure 3: Empirical distribution of absolute difference of velocities ω, where families are restricted to dyads with at least one member under 15.

Table 2: Binary-by-event C_{ij}^{be}, hierarchical stage-1, families with children, $\alpha = 1$ (in %) with detailed confusion rates.

		Work	Leisure
Ground truth	Colleagues	**74.13**	25.87
	Families	15.43	**84.57**
	Couples	16.66	**83.34**
	Friends	39.50	**60.50**

EMD

Table 3: Earth mover's distance C_{ij}, hierarchical stage-1, families with children (in %).

		Work	Leisure
Ground truth	Work	**80.57**	19.43
	Leisure	41.80	**58.20**

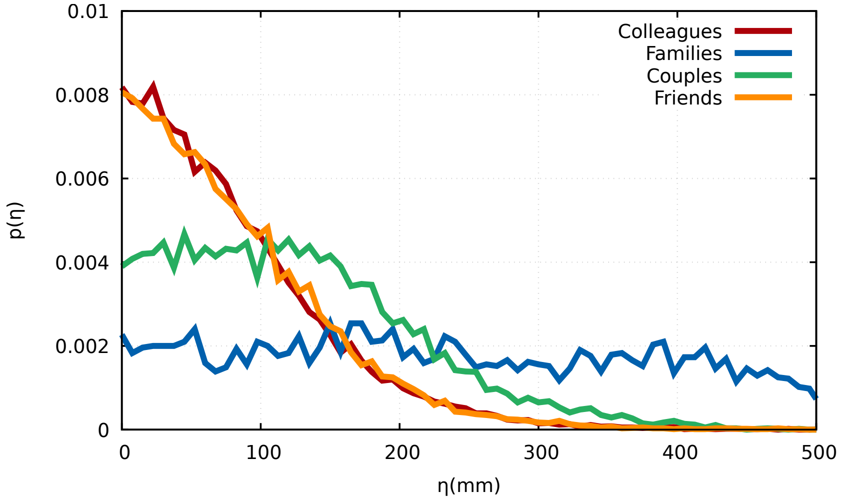

Figure 4: Empirical distribution of height difference of peers η, where families are restricted to dyads with at least one member under 15.

Table 4: Earth mover's distance C_{ij}, hierarchical stage-1, families with children (in %) with detailed confusion rates.

		Work	Leisure
Ground truth	Colleagues	**80.57**	19.43
	Families	39.50	**60.50**
	Couples	28.00	**72.00**
	Friends	46.50	**53.50**

Hierarchical stage-2

Bayesian approach

Table 5: Binary-by-event C_{ij}^{be}, hierarchical stage-2, families with children, $\alpha = 1$ (in %).

		Families	Couples	Friends
Ground truth	Families	**52.51**	24.39	22.80
	Couples	12.50	**45.13**	42.38
	Friends	4.94	18.93	**76.13**

EMD

Table 6: Earth mover's distance C_{ij}, hierarchical stage-2, families with children (in %).

		Families	Couples	Friends
Ground truth	Families	**66.10**	21.80	12.10
	Couples	4.46	**62.94**	32.60
	Friends	1.42	29.05	**69.53**
