# Supplementary material for: Identification of social relation within pedestrian dyads
Source: PLoS One. 2019 Oct 17;14(10):e0223656. doi: 10.1371/journal.pone.0223656 (PMC6797107; doi:10.1371/journal.pone.0223656)
Supplement: S7 Appendix — We report, for the non-hierarchical, α = 1 case, recognition rates obtained by using training sets corresponding to 15% and 50% of the data set. (PDF) [file pone.0223656.s008.pdf]

Recognition with varying sizes of training set

When splitting the data set into training and testing subsets, we want to obtain a training set that is as small as possible, in order to (i) test the ability of our method to be trained on a relatively small set; and (ii) test the stability of our method under variation of the training set (since we repeat different runs using a randomly chosen training set; the smaller is the size of such set, the larger is its variation between different runs).

The choice of using 30% of the data for training was based on considerations on the minimum size that would allow us to build reliable empirical pdfs, i.e. large enough to represent typical behavior (and to satisfy technical requirements such as not having too many empty bins in the empirical pdfs).

To better justify our choice, we performed an analysis concerning the recognition experiments for all the conditions reported in the manuscript (i.e. evaluation measure and α, various definition of families etc.) using also a smaller and larger training set, corresponding respectively to 15% and 50% of the data set. The former is exactly half of what is used to obtain the results in the manuscript, and the latter addresses an equal splitting of the data set.

In what follows, for the sake of brevity, we present the results concerning only the non-hierarchical, $\alpha = 1$ case, which is representative of the overall tendency.

Table 1: **Binary-by-event C_{ij}^{be}, non-hierarchical, $\alpha = 1$ for training ratio of 15%.**

	Colleagues	Families	Couples	Friends
Colleagues	**66.96**	9.48	4.80	18.76
Families	18.82	**41.18**	16.91	23.10
Couples	15.21	**37.74**	25.55	21.50
Friends	34.31	18.60	11.76	**35.33**

Table 2: **Binary-by-event C_{ij}^{be}, non-hierarchical, $\alpha = 1$ for training ratio of 30%.**

	Colleagues	Families	Couples	Friends
Colleagues	**68.31**	7.29	5.37	19.03
Families	18.10	**38.92**	20.66	22.32
Couples	13.58	31.11	**36.57**	18.75
Friends	34.19	16.66	12.74	**36.41**

We may see that, while in general the increase of the training rate to 50% improves recognition rates, the impact is not extremely significant. On the other hand, decreasing the rate to 15% has a negative effect, and for instance the method fails to attain maxima on the diagonal in the C_{ij}^{be} case.

Table 3: **Binary-by-event C_{ij}^{be}, non-hierarchical, $\alpha = 1$ for training ratio of 50%.**

	Colleagues	Families	Couples	Friends
Colleagues	**70.29**	6.27	5.44	18.00
Families	19.84	**36.73**	22.84	20.58
Couples	13.83	25.70	**42.60**	17.87
Friends	34.43	14.78	14.45	**36.35**

Table 4: **Earth mover's distance C_{ij}, non-hierarchical for training ratio of 15%.**

	Colleagues	Families	Couples	Friends
Colleagues	**71.72**	3.02	8.77	16.49
Families	22.00	24.50	**32.91**	20.59
Couples	17.84	12.31	**53.36**	16.49
Friends	**35.78**	6.80	23.88	33.53

Table 5: **Earth mover's distance C_{ij}, non-hierarchical for training ratio of 30%.**

	Colleagues	Families	Couples	Friends
Colleagues	**71.63**	2.88	8.90	16.60
Families	22.23	24.07	**35.22**	18.48
Couples	17.80	11.97	**56.14**	14.09
Friends	**35.62**	5.49	26.28	32.60

Table 6: **Earth mover's distance C_{ij}, non-hierarchical for training ratio of 50%.**

	Colleagues	Families	Couples	Friends
Colleagues	**73.04**	2.79	7.89	16.28
Families	23.09	22.32	**34.81**	19.78
Couples	17.12	11.76	**56.92**	14.20
Friends	**36.62**	4.84	25.24	33.29

Table 7: **Binary-by-trajectory-voting C_{ij}^{v}, non-hierarchical, $\alpha = 1$ for training ratio of 15%.**

	Colleagues	Families	Couples	Friends
Colleagues	**70.85**	7.76	4.06	17.32
Families	18.39	**44.54**	15.31	21.76
Couples	16.02	**35.15**	27.46	21.36
Friends	**39.04**	15.41	11.44	34.11

Table 8: **Binary-by-trajectory-voting C^v_{ij}, non-hierarchical, $\alpha = 1$ for training ratio of 30%.**

	Colleagues	Families	Couples	Friends
Colleagues	**73.18**	5.93	4.59	16.30
Families	19.02	**41.01**	19.40	20.57
Couples	15.20	26.69	**39.89**	18.23
Friends	**39.88**	13.84	12.66	33.63

Table 9: **Binary-by-trajectory-voting C^v_{ij}, non-hierarchical, $\alpha = 1$ for training ratio of 50%.**

	Colleagues	Families	Couples	Friends
Colleagues	**74.27**	5.07	4.68	15.98
Families	20.40	**38.32**	21.85	19.43
Couples	14.08	20.84	**47.16**	17.92
Friends	**38.75**	12.47	14.99	33.79
